# Physically intelligent autonomous soft robotic maze escaper

**DOI:** 10.1126/sciadv.adi3254

**Published:** 2023-09-08

**Authors:** Yao Zhao, Yaoye Hong, Yanbin Li, Fangjie Qi, Haitao Qing, Hao Su, Jie Yin

**Affiliations:** ^1^ Department of Mechanical and Aerospace Engineering, North Carolina State University, Raleigh, NC 27695, USA.; ^2^ Joint NCSU/UNC Department of Biomedical Engineering, North Carolina State University, Raleigh, NC 27695; University of North Carolina at Chapel Hill, Chapel Hill, NC 27599, USA.

## Abstract

Autonomous maze navigation is appealing yet challenging in soft robotics for exploring priori unknown unstructured environments, as it often requires human-like brain that integrates onboard power, sensors, and control for computational intelligence. Here, we report harnessing both geometric and materials intelligence in liquid crystal elastomer–based self-rolling robots for autonomous escaping from complex multichannel mazes without the need for human-like brain. The soft robot powered by environmental thermal energy has asymmetric geometry with hybrid twisted and helical shapes on two ends. Such geometric asymmetry enables built-in active and sustained self-turning capabilities, unlike its symmetric counterparts in either twisted or helical shapes that only demonstrate transient self-turning through untwisting. Combining self-snapping for motion reflection, it shows unique curved zigzag paths to avoid entrapment in its counterparts, which allows for successful self-escaping from various challenging mazes, including mazes on granular terrains, mazes with narrow gaps, and even mazes with in situ changing layouts.

## Teaser

A self-rolling soft robot can autonomously navigate and solve multichannel mazes without the need of human-like brain.

## INTRODUCTION

Achieving autonomy and intelligence in soft robotics is highly desirable yet challenging. These capabilities can substantially reduce the human efforts and control burden during active and adaptive interactions between soft robots, humans, and the environment (*1*–*5*). To achieve autonomy, one common approach is to integrate onboard power supplies [e.g., batteries (*6*–*9*), air pumps (*10*–*12*), or reservoir of compressed gas (*13*)], controls [e.g., microcontrollers (*6*, *7*, *9*, *14*–*16*) and mechanical logic valves (*10*, *13*, *17*)], and sensors [e.g., pressure or tactile sensors (*13*)], to enable controllable actuation and motion. Recent studies have explored integrating tactile sensors with electronics-free pneumatic circuits in autonomous soft-legged robots, taking a step toward intelligent mobile robots capable of reverting walking gaits (*13*). However, these integrations can markedly increase the payloads and complexity of soft robotics. To remove onboard integrations, one strategy is to use remote magnetic or light-responsive actuation for steering untethered soft robotic locomotion. However, these methods require external assistance in manipulating magnetic fields (*18*–*22*) or light sources (*22*–*28*) to guide passive motion, making full autonomy challenging to achieve.

Another potential strategy for autonomy is harvesting the ubiquitous thermal energy from the environments for simultaneous powering and actuation. Soft robots made of thermal-responsive active materials, such as liquid crystal elastomers (LCEs) (*29*–*31*), hydrogels (*32*), and nylon fibers (*33*), can absorb heat or light from the surroundings to perform simple directional autonomous locomotion, such as rolling (*29*, *31*), crawling (*34*), and swimming (*32*). However, while capable of autonomous motion, they lack intelligence as they cannot self-adapt to complex unstructured environments with obstacles, limiting their abilities in complex adaptive motion and self-navigation, such as navigating through mazes.

Maze navigation and solving serve as important tools in biology and robotics to evaluate decision-making and intelligence levels. For example, T mazes and radial arm mazes are commonly used to train and test the learning, memory, and other behaviors of small animals in neurosciences (*35*–*38*). In the realm of artificial intelligence and machine learning, a robot’s ability to escape mazes is believed to reflect its intelligence level (*39*–*42*). However, to date, soft robotic maze escaping has primarily relied on human manipulation rather than autonomous self-solving (*16*, *43*–*46*).

Instead of relying on neuron-based computation intelligence in the brain, an alternative paradigm known as physical intelligence (PI) has emerged for designing autonomous and intelligent soft robots (*4*, *47*). PI harnesses both materials intelligence from smart materials and mechanical intelligence from novel structural designs to achieve self-sensing, actuation, control, adaption, and decision-making without external computation or sensors (*4*, *47*). Recently, we have reported a twisted LCE ribbon–based soft self-rolling robot that autonomously escapes from simple obstacle course–like confined spaces (*48*), e.g., an enclosed wall with a central diamond-shaped island and an exit. When encountering an obstacle, the robot can adaptively react in two ways depending on the location of the interaction contact point. It can either passively turn around the obstacle or reflect its self-rolling motion via snapping (*48*). This highlights the potential of using PI without any human intervention or external controls in achieving autonomous navigation in unstructured environments for soft robots.

Despite advances, susbstantial limitations persist when dealing with more complex unstructured environments, such as mazes with multichannel confined spaces, which poses a great challenge for soft mobile robots due to high requirements. To escape from mazes, fundamental strategies and requirements must be met, including the need for active self-turning capability, self-adjustment in paths, and trial-and-error searching. It requires the robot to make active turns for motion redirection when confronted with walls and branches, self-adjust motion pathways to avoid trapped in channels or loops, and rely on massive trial-and-error search routes to cover more areas until the correct pathways are found. However, our previous twisted LCE ribbon robot fails to satisfy these requirements (*48*), because it lacks one of the most important active and sustained self-turning capabilities. As a result, it becomes permanently trapped even in a simple confined space composed of two parallel walls, a representative scenario in a typical maze, as it keeps bouncing back and forth between the two walls on the same spot through snapping (fig. S1A and movie S1). Consequently, it hinders self-adjustment for escaping traps and trial-and-error searching, making autonomous maze escaping impossible. Thus, a new solution is needed to address the challenge for achieving an autonomous and intelligent soft robotic maze escaper. Moreover, compared to rigid robotic maze escapers, the advantage of active and adaptive interactions with environments in soft robots remains unexplored during maze navigation, especially in more challenging mazes for rigid robots that require active shape and dynamic environmental adaptability, such as mazes with narrower gaps than robot body sizes and dynamic mazes with in situ changing maze layouts.

Here, we report harnessing geometric intelligence in a library of hierarchically twisted LCE-based soft robotic rollers for self-navigation in complex mazes. We demonstrate that manipulating the geometric asymmetry in the soft robot with hybrid twisted and helical shapes on two ends enables built-in active and sustained self-turning capabilities, without sacrificing adaptive robot-environment interactions via snapping for path reflection, while its two geometrically symmetric counterparts with either twisted or helical shapes undergo transient self-turning. Through experiments, numerical simulation, and analytical modeling, we explore the underlying mechanism underpinning the self-turning behaviors. The combination of built-in sustained self-turning and self-snapping enables self-adjustment in motions and large area coverage for trial-and-error searching, facilitating successful self-escaping from both simple parallel confined spaces and complex multichanneled mazes and even mazes in granular terrains. Moreover, it showcases self-navigation through more challenging mazes with narrower gaps than its body size via active shape adaption and dynamic mazes with in situ changing patterns.

## RESULTS

### Fabrication of hierarchically twisted LCE ribbons

**Fig. 1.**
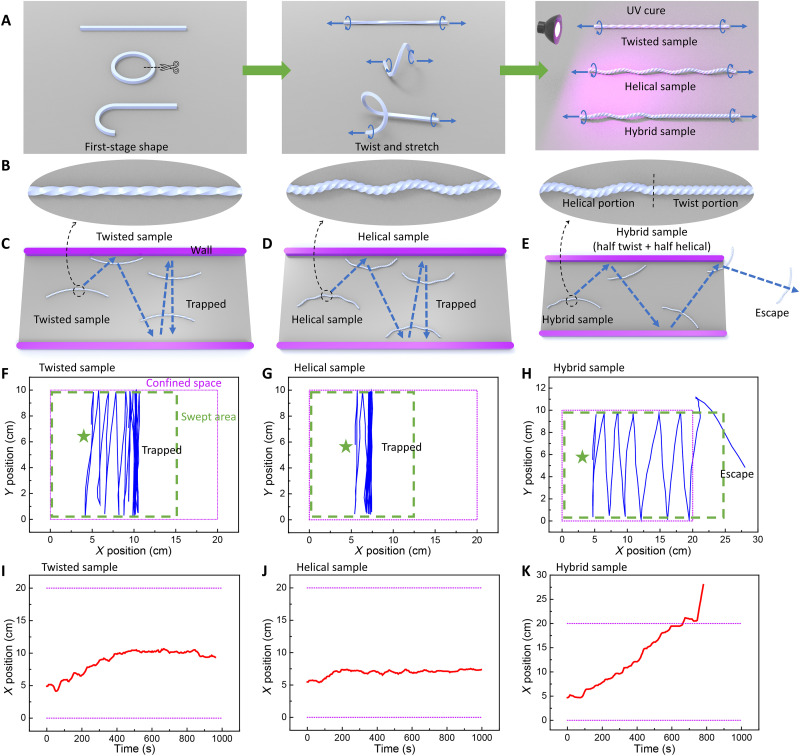
Self-escaping performances of the twisted, helical, and hybrid twist-helical LCE ribbons from a simple parallel confined space on a hot surface. (**A** and **B**) Schematics of two-step fabrication of the three samples by stretching and twisting a straight, circular, and “J”-shaped ribbon during UV curing, respectively. The hybrid sample has equal length of the helical and twisted portion. (**C** to **E**) Schematics of their motion paths within two parallel walls (20-cm length and 10-cm spacing) with their experimental paths shown in fig. S1. The three samples’ trajectories (**F** to **H**) and horizontal displacements (*X* positions) evolving with time (**I** to **K**), respectively. Star symbols denote the starting points. The purple boxes denote the boundary of the confined space. The green dashed boxes denote the areas swept by the samples. The hot surface temperature is 120°C.

Figure 1A shows the schematics of fabricating three twisted and hierarchically twisted LCE ribbons without and with geometric asymmetry. The LCE is synthesized with modified thiol-acrylate Michael addition reaction method in two-stage polymerization (*49*). The three samples take different first-stage–cured shapes such as a straight (length, 60 mm), circular (radius, 5 mm), and J-shaped ribbon (composed of a half semicircle with radius of 5 mm and a half-straight ribbon), respectively, while they have the same square cross sections with a side length of 2.5 mm (note S1 and fig. S2). During the second-stage curing with ultraviolet (UV) light, all the samples were stretched (stretching strain ɛ = 100%) and twisted (twist density = 0.5 turn/mm) to straighten the ribbons and align the mesogens with the stresses (*48*). The three samples take the postfabricated shapes of a twisted ribbon, a helical ribbon with hierarchically twisted features, and a hybrid helical-twist ribbon with the same twisting chirality (left-handedness), respectively (see fig. S1, A and B, for details). We find that the helical ribbon exhibits a two-order hierarchical structure that contains higher-order helical waves and lower-order twists, where both diameter and wave pitch length are inversely proportional to the twist density (fig. S3). The hybrid ribbon is composed of a half-hierarchical helical-twist portion and a half-twisted portion, which breaks the geometric symmetry in its two counterparts.

### Self-escaping capability in parallel confined space

The ability of escaping from parallel confined space is essential for complex maze escaping, because the complex mazes can be segmented into multiple zones confined with parallel walls. Next, we examine their autonomous motion and self-escaping capabilities in a simple confined space composed of two parallel walls (length ~ 20 cm and spacing ~10 cm) on a hot surface (120°C). As shown in Fig. 1 (C to E), fig. S1, and movie S1, the three ribbons were initially placed in parallel to the walls at the same starting points. Similar to the twisted ribbon, the two helical and hybrid ribbons can continuously self-roll on the hot surface with their centerlines slightly bended toward the rolling direction driven by the thermal gradient along the ribbon width (*48*), as well as flip the rolling direction when meeting the walls via passive self-snapping (*48*). We note that both the twisted ribbon and the helical ribbon were trapped in the channel by continuously reflecting between the two walls. In contrast, the hybrid ribbon was capable of self-escaping after 12 trials of snapping within 1000 s. Similar self-escaping phenomenon can be observed in a randomly angled hybrid ribbon while its two counterparts were trapped inside the channel (figs. S4 and S5).

Figure 1 (F to H) shows the trajectories of the three ribbons with the time-evolving *X*-coordinates (i.e., horizontal movement) shown in Fig. 1 (I to K). In the beginning, i.e., from 0 to 200 s, all the ribbons follow a similar zigzag reflection path and move slightly rightward with an increasing *X*-coordinate. We note that the zigzag motion path is due to the active self-turning behavior of the three ribbons during self-rolling away from the walls. The slight turning induces an angle of incidence when the ribbons meet the walls. The straight wall can always align all the different angled ribbons against the wall via the adaptive ribbon-wall interaction irrespective of their incident angles and then reflect them normal to the wall via self-snapping (fig. S1 and movie S1). This also holds true for all the ribbons initially placed in a random angle within the channel (figs. S4 and S5 and movie S1). However, for both the twisted and helical ribbons, after a few trials of snapping and interactions with the wall (12 and 4 trials for the twisted and helical ribbons, respectively), their zigzag paths transit to a repeated and overlapping I-shaped path by reflecting the ribbons normal to the walls with a zero incident angle, indicating that the two ribbons roll straightly with their self-turning capabilities vanished after reaching thermal equilibrium (note S2 and fig. S6). Meanwhile, their time-evolving *X*-coordinates approach a plateau, and the two ribbons were trapped inside the channel. In sharp contrast, for the hybrid ribbon irrespective of the placed angles, it always preserves the zigzag reflection path with a linearly increasing *X*-coordinate until escaping the channel. The zigzag path also enables the hybrid sample to cover larger areas (~1.67 and ~2 times larger than the swept areas of the twisted and helical ribbons, respectively) in a shorter duration, which favors the fundamental maze escaping strategies (Fig. 1, F to H). This is attributed to its intrinsic superior active self-turning capability induced by the geometric asymmetry.

### Active self-turning behavior during unconstrained self-rolling

The self-escaping capabilities in a confined space are mainly determined by the active self-turning behavior when the ribbons move away from the walls because the walls can always snap and reflect the ribbons normal to the wall irrespective of the incident angles. To better understand their self-escaping capabilities, next, we study the active self-turning behaviors of the three ribbons during free self-rolling on a hot surface without obstacles.

**Fig. 2.**
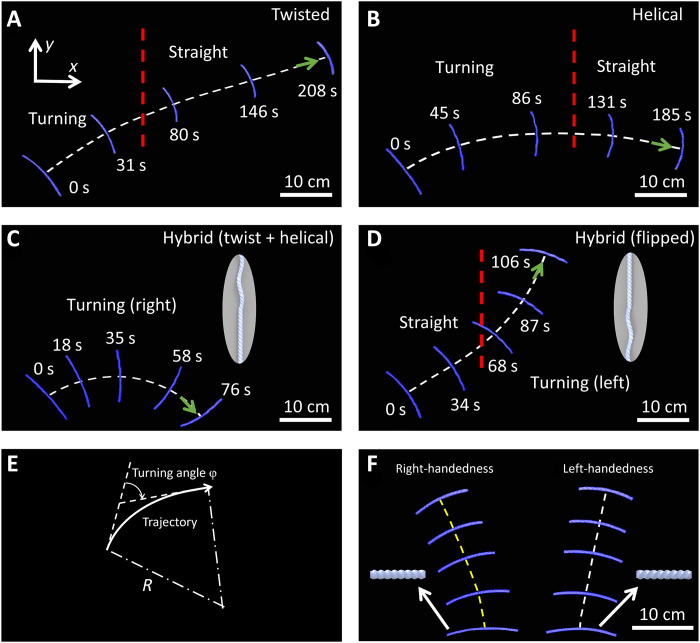
Unconstrained self-rolling and self-turning behavior of the twisted, helical, and hybrid ribbons on a hot surface. (**A** and **B**) The overlapped time-lapse trajectories for the twisted (A) and helical (B) samples. (**C** and **D**) The overlapped time-lapse trajectories for the same hybrid sample before (C) and after flipping placement (D). Insets show the unflipped and flipped configurations. The red-color dashed lines denote the path transition from a straight to a curved path or vice versa. (**E**) Schematic for the turning angle ϕ and turning radius *R*. Right turn is defined as positive. (**F**) Comparison of the overlapped trajectories in the twisted ribbon with opposite handedness. The hot surface temperature is 120°C.

**Fig. 3.**
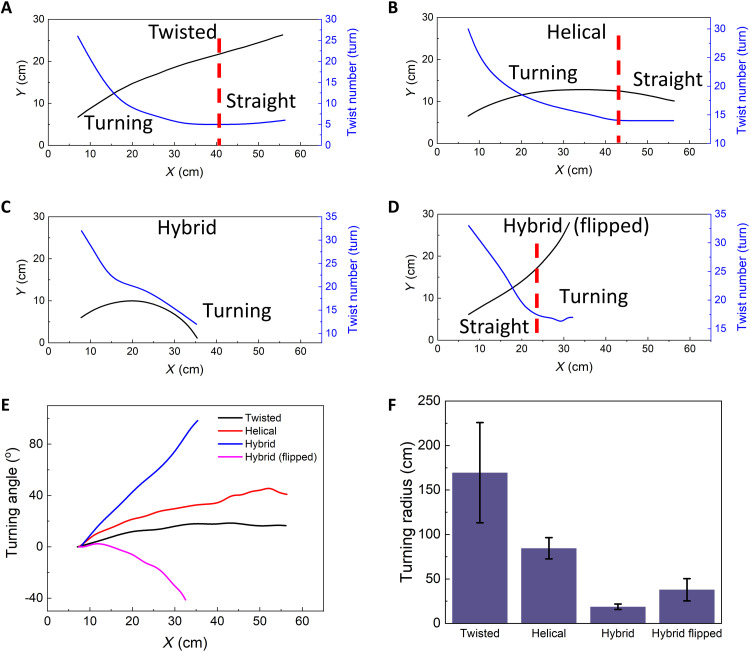
Self-turning behavior characterization. (**A** to **D**) The measured trajectories and instantaneous twist numbers in the twisted ribbon (A), the helical ribbon (B), and the hybrid ribbon before (C) and after flipping (D). The red-color dashed lines in (A), (B), and (D) denote the path transition from a curved to a straight path or vice versa. (**E**) The self-turning angle versus *X* position in different ribbons. (**F**) The self-turning radius in different ribbons.

Figure 2 (A to D) shows the overlapped time-lapse motion images of the respective three twisted, helical, and hybrid ribbons with the same left-handedness when placed on a large hot surface (120°C) at the same starting location (movie S2). Specially, for the hybrid ribbon, considering its geometric asymmetry along the axis, it can be either placed with its helical part to the left as shown in Fig. 2C or flipped with its helical part to the right as shown in Fig. 2D. The corresponding tracked motion trajectories alongside self-turning angle and radius (Fig. 2E) are shown in Fig. 3.

Both the twisted ribbon and the helical ribbon exhibit an initial right turning arc-shaped path followed by a straight-line path (Figs. 2, A and B, and 3, A and B), attributed to the transient untwisting in the beginning and the cease of untwisting before and after reaching thermal equilibrium, respectively, as discussed next. We note that such untwisting-induced transient active self-turning is universal, as evidenced by the finite element simulation discussed later and not reported in previous studies of autonomous soft rollers (*29*, *31*, *33*, *48*). Thus, the self-turning capabilities in both ribbons are not sustainable. In contrast to the transient self-turning in its two counterparts, the hybrid ribbon demonstrates self-sustained, steady-state, and asymmetric left-/right-turning capabilities. Figures 2C and 3C show that it keeps self-turning to the right with a continuous arc-shaped path, whereas its flipped case self-rolls along a straight line in the beginning and then transits to continuous left turning (Figs. 2D and 3D) before and after reaching thermal equilibrium, respectively.

We note that the self-turning direction induced by untwisting is intrinsically determined by the twisting chirality. Figure 2F and fig. S7 show that the two twisted ribbons with opposite chirality follow a mirrored self-rolling trajectory, where the right-handed one prefers left (counterclockwise) turning in the beginning followed by linear motion, as opposed to right turning (clockwise) in the left-handed one. The turning direction is independent of its rolling directions and placement orientations (e.g., flipped or redirected).

To characterize their self-turning behavior, we plot the evolution of the turning angle ϕ during self-rolling motion in Fig. 3E, where ϕ is defined as the tangential angle with respect to the tangential line at its original position along the path (Fig. 2E, right turning is defined as positive). For both the twisted ribbon and the helical ribbon, ϕ increases first and then approaches to a plateau, where the helical ribbon shows a twice larger turning angle of ~40^o^ than the twisted one. In contrast, for the hybrid ribbon, ϕ increases monotonically to a large value of over 90^o^, which is over twice larger than its left-turning flipped counterpart (ϕ ~ −40^o^). The turning radius *R* defined as 1/*R* = dϕ/d*s* (*s* is the arc length; Fig. 2E) describes the variation of ϕ along the path, which characterizes self-turning capability. A larger dϕ/d*s* leads to a smaller *R*, i.e., stronger self-turning capability. Among all the ribbons, the right-turning hybrid ribbon shows the strongest turning capability with the smallest *R* = 19 cm, which is nine times smaller than the twisted one with the largest *R* = 170 cm (Fig. 3F). For the same hybrid ribbon, its right-turning radius *R*_R_ is 2 times smaller than the left-turning radius *R*_L_, showing stronger right-turning capability than left-turning. The large error bars are due to the inhomogeneous temperature distributions of the hot plate (fig. S8).

**Fig. 4.**
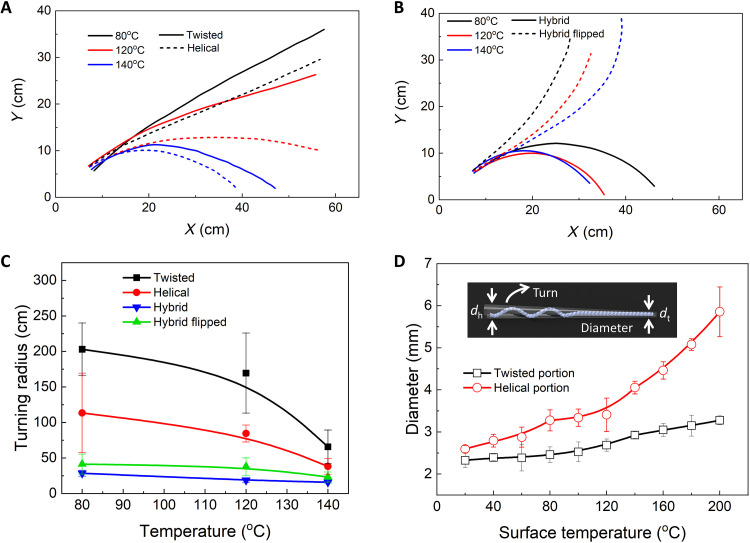
Surface temperature effects on the active self-turning behavior of the twisted, helical, and hybrid ribbons. (**A**) The trajectories of the twisted (solid curves) and the helical (dashed curves) ribbons at different surface temperatures. (**B**) The trajectories of the hybrid ribbons before (solid curves) and after flipping (dashed curves) at different surface temperatures. (**C**) The turning radius as a function of surface temperatures for all the ribbons. (**D**) The diameter change of the two twisted and helical ends in the hybrid ribbon with surface temperatures. Inset shows the schematic of a conical-like shape in the hybrid ribbon with different diameters on two ends for active turning.

Next, we explore the effect of the surface temperature *T* within autonomous rolling temperature range (i.e., 55° to 220°C) on their trajectories. Figure 4 (A and B) shows that similar trajectory profiles to that at *T* = 120°C are also observed at *T* = 80° and 140°C for each ribbon type (twisted, helical, hybrid, and hybrid flipped). As *T* increases from 80° to 140°C, for all the ribbon types, the turning angle ϕ also increases (fig. S9), resulting in a more curved profile (Fig. 4, A and B) with a decreasing turning radius *R* (Fig. 4C), demonstrating enhanced self-turning capabilities at a higher *T*. For all different *T*, the hybrid ribbon without flipping shows the smallest *R*, while the twisted ribbon shows the largest *R* (Fig. 4C), corresponding to the strongest and weakest self-turning capability, respectively.

### Active self-turning and motion mechanism

To better understand the self-turning behavior and different motion trajectories in the self-rolling ribbons, next, we combine experiments, finite element analysis (FEA) simulation, and analytical modeling to exploit their underlying mechanisms.

As shown in Fig. 3 (A and B), we find that self-turning in both the twisted ribbon and the helical ribbon is mainly attributed to the untwisting process, arising from the temperature elevation–induced phase transition of the LCEs (*48*). Such untwisting is also accompanied by the coupled shrunk length and enlarged cross sections (figs. S10 and S11). Both the twist number (Fig. 3, A and B) and the ribbon length (fig. S10, A and B) exponentially reduce first, then approach to a plateau, and become stabilized without untwisting and size change after reaching thermal equilibrium (*48*), leading to a linear motion without turning. For example, for the helical ribbon, its twist number reduces from 30 to a stabilized 6, and its length shrinks by ~30% when self-rolling on a hot surface with *T* = 120°C.

**Fig. 5.**
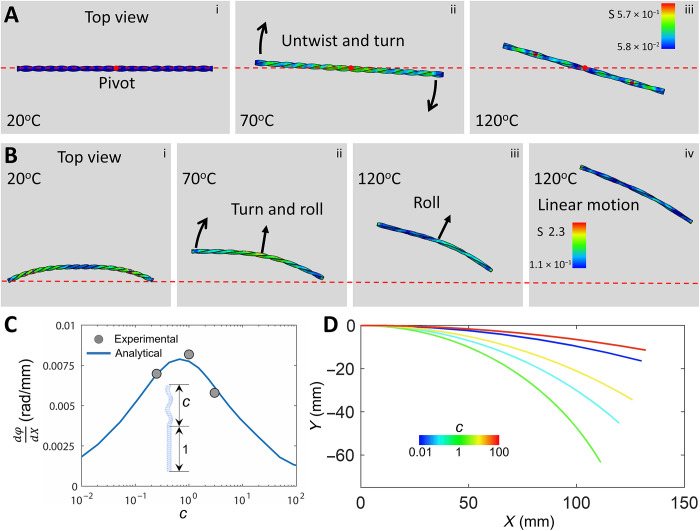
FEA simulation and theoretical analysis on the self-turning behavior. (**A** and **B**) FEA simulation of self-spinning behavior around the central pivot (A) and self-turning behavior during rolling [(B), i to iii] in the twisted LCE ribbon on a frictional surface due to temperature increase–induced untwisting. When untwisting stops, the rolling ribbon follows a linear motion [iii and iv in (B)]. (**C** and **D**) Theoretical analysis on the effect of helical to twisted segment length ratio *c* in the hybrid ribbon on steady-state self-turning behavior in the hybrid ribbon. The comparison between analytical modeling and experiments on turning angle change dϕ/d*X* as a function of ratio *c* (C). (D) The predicted steady-state trajectories of the hybrid ribbons with different ratio *c*.

The untwisting-induced directional self-turning in the rolling twisted LCE ribbon is further validated by the simplified dynamic FEA simulation. For better understanding, we decompose the whole process into two scenarios. First, we simulate a thermal-induced untwisting process in the ribbon without rolling. As shown in Fig. 5Ai and movie S3, the left-hand twisted LCE ribbon is placed on a frictional rigid surface. It ensures surface contact under its body weight. Upon heating the ribbon, it starts to shrink in length and untwist itself. Untwisting makes the ribbon self-spin clockwise around the midpoint of the ribbon because of the asymmetric friction induced torque (Fig. 5A, ii and iii, and movie S3). Second, in addition to thermal-induced twisting, we set the bended ribbon to roll forward. As shown in Fig. 5B, i and ii, the clockwise pivoting about the midpoint combined with the rolling turns into a right turn with the increase of the temperature. Once the temperature does not increase and remains constant, the untwisting stops and the ribbon switches to linear rolling motion without active turning (Fig. 5B, iii and iv, and movie S3). When the chirality is switched to right hand, it self-turns to the left. Meanwhile, as the temperature increases, it untwists more and leads to a smaller turning radius (fig. S12). The simulation well reproduces the experimental results. Thus, the transient self-turning and steady-state linear motion in both the twisted ribbon and the helical ribbon explain the observed trapping in the two parallel walls in Fig. 1 (C and D). It also explains the often-observed linear motion reported in most soft self-rollers using a cylindrical or helical shape due to the lack of untwisting characteristics (*29*, *31*, *33*).

For the hybrid ribbon, in addition to the similar untwisting-induced self-turning, it has one more self-turning mechanism arising from the geometric asymmetry with different nominal diameters at two ends (Fig. 4D and inset). The hybrid ribbon shows an approximately conical shape with a slightly larger diameter *d*_h_ in the helical segment than the twisted one *d*_t_ (*d*_h_/*d*_t_ *~* 1.1 in room temperature; fig. S13). As expected, during self-rolling, such geometric asymmetry will always drive the self-turning around the smaller diameter end, i.e., the twisted end, which can be either clockwise or counterclockwise turning depending on its placement orientation (fig. S14). Such geometric asymmetry-induced self-turning is independent of its chirality. In contrast, the untwisting-induced self-turning direction is independent of its placement orientation and only dependent on its chirality.

The synergy and competition between the geometric asymmetry and chirality-related untwisting-induced self-turning determine the shape of its motion path. For example, for a left-handed hybrid ribbon, when its thinner twisted end is placed on the right, both the geometric asymmetry and the untwisting induce the same clockwise self-turning, which leads to enhanced right-turning capability with a smaller turning radius as shown in Figs. 2C and 3C. In contrast, when it is flipped with the twisted end on the left, the geometric asymmetry induces counterclockwise self-turning, whereas the untwisting induces clockwise self-turning. Thus, the self-turning could become canceled in the beginning, which leads to the initial linear motion as shown in Figs. 2D, 3D, and 4B. After approaching the thermal equilibrium, the untwisting nearly stops. Consequently, clockwise turning ceases and the self-turning is mainly governed by the geometric asymmetry for counterclockwise turning, which accounts for the followed left-turning with a larger turning radius as shown in Figs. 2D, 3D, and 4B. As *T* increases, more untwisting leads to a reducing turning radius *R* for all the ribbons (Fig. 4, A to C). For the hybrid case, *d*_h_/*d*_t_ increases with *T* (Fig. 4D and fig. S13), which enhances the geometric asymmetry. Consequently, when combining with untwisting, it results in a further reducing *R* at higher *T* observed in Fig. 4C.

### Geometric effects on active self-turning and self-snapping behaviors

Furthermore, we first explore programming the turning capability by tuning the length ratio *c* of the helical to twisted segment in the hybrid ribbon. *c* = 0, 1, and ∞ correspond to the studied three special cases: a twisted ribbon, a hybrid ribbon with half-twisted and half-helical, and a helical ribbon, respectively. Theoretically, after reaching thermal equilibrium, the variation in the turning angle ϕ along the *x*-axis displacement *X* takes the form of (note S3)

$$\frac{d\varphi }{dX}=\frac{2\sigma (d_{h}-d_{t})(1+c)}{l\sigma_{\max}(cd_{h}+d_{t})}$$ (1)

where σ/σ_max_ denotes the scattering of the velocity of the line elements along the hybrid ribbon and *l* is the ribbon length. Figure 5C shows the predicted *d*ϕ*/dX* as a function of *c* (fig. S15). As *c* increases, *d*ϕ*/dX* increases first and then decreases. At *c* = 1, it shows a peak value, denoting a critical point for the largest scattering in the velocity of the line elements in the hybrid ribbon. It leads to the largest velocity variation in the escaper accompanied by the largest turning angle per displacement along the *x* axis. This is consistent with the experiments (Fig. 5C) and the predicted steady-state trajectories as a function of *c* (Fig. 5D). It shows that the trajectory of the hybrid ribbon with *c* = 1 has the smallest turning radius (Fig. 5D), indicating the strongest turning capability that facilitates the maze escaping.

Thus, equipped with the geometric asymmetry induced continuous active self-turning capability, the hybrid ribbon can always self-escape from the confined space of two parallel walls in Fig. 1H by following the zigzag path with the help of snapping (note S4, figs. S16 and S17, and movies S4 and S5). The zigzag path is due to the switch of its self-turning directions during back-and-forth rolling: When the left-handed hybrid ribbon with its twisted end on the right in Fig. 1H self-rolls toward the top wall, it self-turns to the right. However, once self-rolling away from the top wall after aligning and snapping, it self-turns to the left. Thus, the continuous zigzag turning makes it move toward the right end for exiting. We note that in addition to the active self-turning, when either end of all the ribbons is blocked by an obstacle, they can passively turn around it for changing directions (fig. S18).

Next, we further explore the effect of the geometric size of the hybrid ribbon on its self-turning and self-snapping behavior. We vary the side length *a* of the square cross section and the ribbon length *l*, with *a* ranging from 1 to 5 mm and *l* varying from 5 to 12 cm (note S5). In general, most hybrid ribbons of different dimensions exhibit similar motions, including self-rolling, active and passive self-turning, and/or self-snapping. However, each ribbon exhibits different behaviors concerning the working temperature for both self-turning and self-snapping motions. For example, ribbons with the same cross-sectional size (*a* = 2.5 mm) but different lengths show that shorter ribbons (e.g., *l* = 5 cm) tend to snap rather than passively turn even when one end is blocked by an obstacle because of the sacrifice of turning moment (fig. S19A). On the contrary, longer ribbons (e.g., *l* = 12 cm) preferred passive turning when one end is blocked by an obstacle (fig. S19B). Similarly, ribbons with the same length (*l* = 10.5 cm) but different cross-sectional sizes reveal that ribbons with smaller *a* (e.g., *a* = 1 mm) cannot sustain self-rolling on hot surfaces when the temperature changes from 50° to 220°C. This is because the small driving force makes the sample highly sensitive to defects during motion and become stuck when it becomes homogeneously heated, losing the thermal gradient across the cross section. Increasing *a* results in the ribbon tending to snap when one end is blocked by an obstacle (e.g., *a* = 5 mm; fig. S19C). Figure S20 shows the temperature ranges wherein the ribbons can snap or make passive turns. The overlapping regions indicate their working temperatures, i.e., the ribbons can both snap and turn for self-escaping from mazes. The ribbon with *a* = 2.5 mm and *l* = 10.5 cm shows relatively low working temperatures and a large temperature range, making it an ideal model system for exploring self-escaping behaviors in different mazes unless otherwise specified.

### Autonomous escaping from mazes

**Fig. 6.**
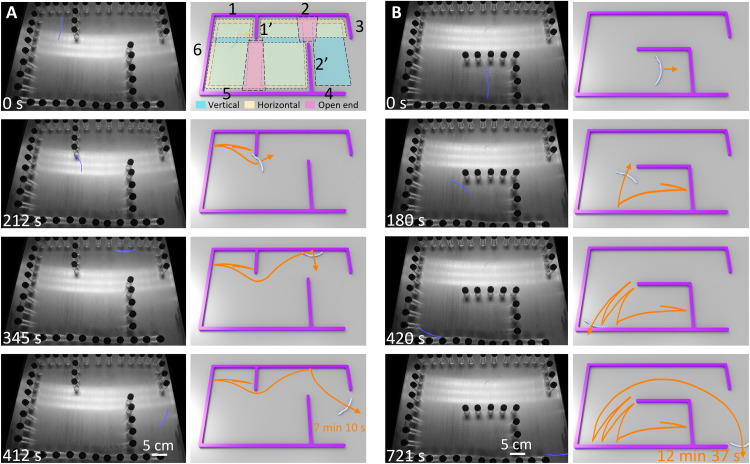
Self-escaping of the hybrid ribbon from simple mazes with two different layouts of two partition walls on a hot surface. (**A** and **B**) Selected time-lapse images during self-escaping from mazes with two parallel partition walls (A) and two right-angled walls (B) made of aligned glass bottles. In (A), two partition walls divide the maze into six horizontal (yellow) and vertical (blue) channeled subspaces to potentially trap the ribbon. Exit is on the bottom right. The starting points are randomly selected away from the exit. Right column shows the corresponding schematics of trajectories in experiments. Related video is shown in movie S6. The hot surface temperature is 120°C.

**Fig. 7.**
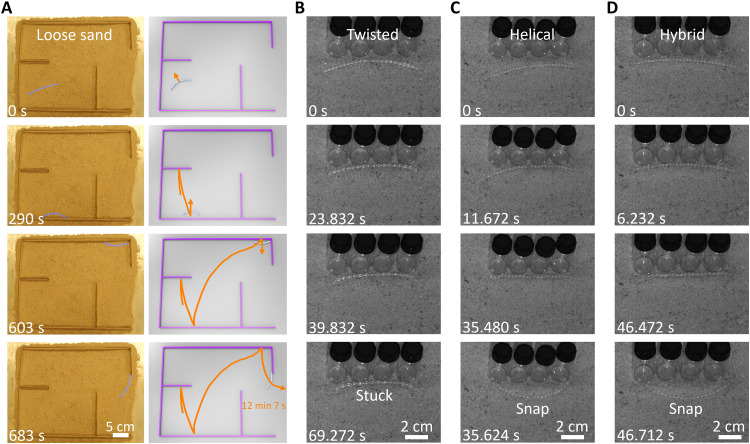
The snapping and self-escaping of the hybrid ribbon from simple mazes on loose sand. (**A**) Selected time-lapse images during self-escaping from a maze on sand with two perpendicular walls made of wooden plates. The schematics are shown on the right. (**B**) The twisted ribbon is stuck in sand and cannot snap-through. (**C** and **D**) The snap-through processes of the helical (C) and hybrid (D) samples on sand. The hot surface temperature is 120°C.

**Fig. 8.**
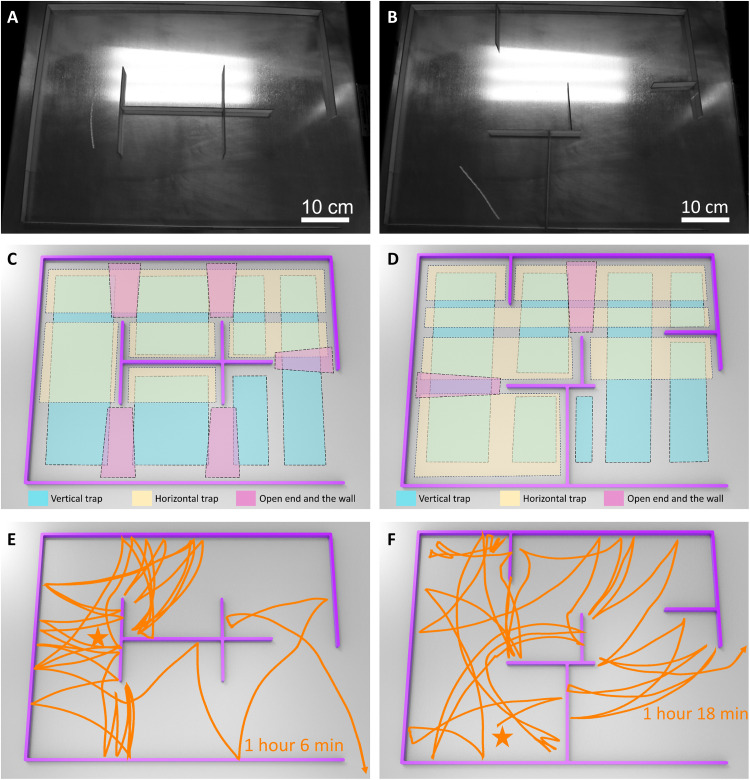
Self-escaping of the hybrid ribbon from complex mazes with two different layouts of six partition walls on a hot surface. (**A** and **B**) Images of maze 1 (A) and maze 2 (B) in movie S9. Six partition walls divide the two mazes into 9 and 12 horizontal and vertical channeled subspaces, respectively. (**C** and **D**) The schematics for the combinations of the subspaces of the maze in (A) and (B). (**E** and **F**) The corresponding escaping trajectories from random starting points (denoted as star symbols) away from the exits in (A) and (B). The hot surface temperature is 120°C.

On the basis of the uncovered active self-turning mechanisms in the self-rolling ribbons, we exploit their intelligent self-escaping capabilities from complex confined spaces such as channeled mazes with different levels of complexity on a hot surface (120°C). As shown in Figs. 6 to 8, the channeled mazes are constructed from adding a few number of partition walls into a rectangular-shaped confined space with an exit on the bottom right. The partition walls are designed to create a certain number of small channeled subspaces that could potentially trap the ribbons inside and increase the difficulty in self-escaping. The distances between walls are set to be larger than the sample length to allow its self-rolling inside. Each sample is randomly placed in the channels for at least three times away from the exit to test their self-escaping capabilities, where their motion trajectories and escaping duration are recorded.

First, we start with simple mazes with fewer partition walls (e.g., lined glass bottles or thin wooden plates) and less channels. Figure 6 (A and B) and fig. S21 show the three designs of a simple maze on a hot surface (120°C) with two partition walls placed in parallel, an L shape, and perpendicular to each other apart, respectively (movie S6). For all the three designs, the two partition walls divide the rectangular-shaped confined space into a total of six horizontal (“=”) and vertical (“||”) channeled subspaces formed by two parallel facing walls without any dividers, labeled by yellow and blue zones in the figures. For example, for the design in Fig. 6A, it has three “||” channeled subspaces formed by the pair of numbered vertical walls of 6-1′, 6–2′, and 1′-3 and three “=” channeled ones composed of horizontal walls of 1-5, 2-5, and 2-4. These channeled subspaces are similar to the aforementioned confined space of two parallel walls shown in Fig. 1, which could potentially trap the self-rolling ribbons inside either horizontal or vertical channels. Furthermore, similar potential traps could occur between the open ends of partition walls of 1′ and 2′ and the opposing walls of 5 and 2, respectively, labeled by purple zones in the figures. As expected, because of the loss of active self-turning capability without untwisting after reaching thermal equilibrium, both the twisted ribbon and the helical ribbon are easy to get trapped inside the channeled subspaces by repeatedly bouncing back and forth between walls via snapping and passive turning for even more than 2 hours (figs. S22 and S23 and movie S7), making it very challenging to self-escape the three simple mazes. In contrast, for the hybrid ribbon, its zigzag curved paths with opposite self-turning directions before and after snapping largely increase the escaping chances. Thus, equipped with such geometric asymmetry–induced continuous active self-turning capabilities, the randomly placed hybrid ribbon in different starting positions of the three mazes can always find its way out for self-escaping by combining with self-snapping and passive turning. Depending on the starting locations and the designs of the maze, its self-escaping duration can vary from about 7 min to close to 1 hour with distinctly simple to complex trajectories (Fig. 6, A and B, figs. S21 and S24, and movie S6).

Similarly, the hybrid ribbon can also self-escape from the simple maze on more challenging granular substrates, e.g., loose sand on a hot surface (120°C) in Fig. 7A and movie S8. Compared to the smooth and rigid hot surface, the granular substrate makes it hard to snap for motion reflection from the wall because of the fluid-like sand particles (note S4, figs. S16 and S17, and movies S4 and S5). We find that the twisted ribbon loses its snapping capability when self-rolling on sand against a wall, because it is easy to get stuck by burrowing its two ends into the sand because of its sharp blade-like boundary, making it very challenging to snap (Fig. 7B and fig. S17D). In contrast, the smooth helix shape of both the helical and hybrid ribbons can help preventing from the sand burrowing, thus exhibiting better snapping performances than the twisted ribbon (Fig. 7, C and D; fig. S17, E and F; and movie S5), while it takes a longer snapping time of 150 to 250 ms than that on rigid surfaces (about 100 ms; figs. S16 and S17). Despite the preserved snapping capability in the helical ribbon, it fails to self-escape from the simple maze on sand because of the lack of continuous active self-turning capability. For the hybrid ribbon, it takes a longer time of about 12 to 50 min to self-escape on sand than on rigid surfaces (Fig. 7A, fig. S25, and movie S8).

Second, to increase the complexities in the maze, we introduce more number of branched partition walls (e.g., six thin wooden plate walls) into the rectangular-shaped confined space. As shown in Fig. 8 (A and B), the two complex mazes have a number of about 11 and 12 divided horizontal and vertical channeled subspaces for potentially trapping the self-rolling ribbons between two walls (Fig. 8, C and D), respectively. In addition, it creates five open ends of the partition walls (purple zones in Fig. 8C) as opposed to one or two open ends in the simple mazes in Figs. 6 and 7. The open ends will facilitate both snapping and passive self-turning of the self-rolling ribbons, depending on their encountering positions (*48*). These open ends further increase the chance of being trapped by bouncing back and forth either between two open ends or between one open end and one wall as shown in Fig. 8D, making the complex mazes more challenging to escape. As expected, only the hybrid ribbon can successfully escape from the two complex mazes with the other two twisted and helical ribbons being permanently trapped. Figure 8 (E and F) shows the tracked self-escaping trajectories in the two complex mazes (movie S9). It takes a much longer self-escaping duration of over 1 hour to escaping from the aforementioned potential trapping scenarios between walls and open ends–walls.

### Autonomous escaping from mazes with narrow gaps or in situ changing layouts

To further challenge its maze-escaping capabilities, we explore more difficult scenarios involving mazes with narrower gaps compared to the soft robot’s body size or undergoing in situ changing layouts. In both cases, the hybrid ribbon–based soft robot is required to actively self-adapt to navigate through tight spaces and dynamically changing environments. This challenge is not easily achievable by rigid robots because of their inflexibility.

**Fig. 9.**
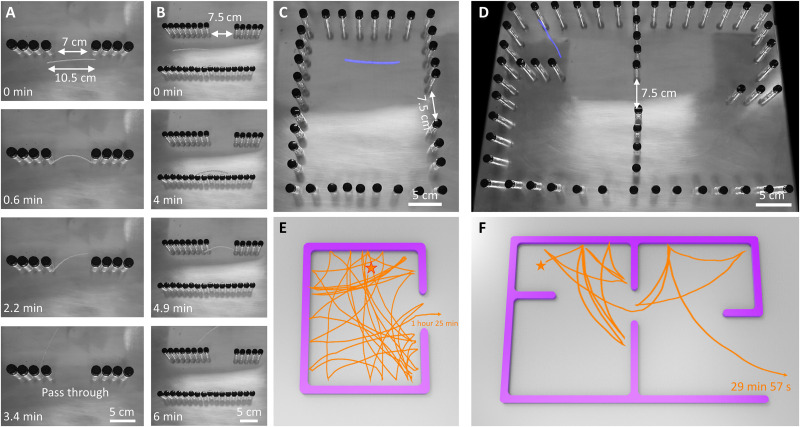
Self-navigation through simple confined spaces and mazes with a narrow gap. (**A**) The self-rolling hybrid ribbon (length, 10.5 cm) passes through a narrow gap (width, 7 cm). (**B**) The ribbon self-escapes from a parallel confined space with a narrow gap. (**C** and **D**) Images of an enclosed confined space with a narrow gap as the exit (C) and a four-chamber maze with a narrow gap between the walls in the middle (D). (**E** and **F**) The corresponding escaping trajectories from random starting points (denoted as star symbols) away from the exits in (C) and (D). The hot surface temperature is 120°C.

Before examining mazes with narrow gaps, we explore the robot’s ability to pass through narrow gaps in two simpler cases. In the first case, we introduce a narrow gap to a single wall, and the robot, when self-rolling toward the center of the gap (Fig. 9A), can pass through gaps as narrow as ~7 cm, about 33.3% smaller than its length of 10.5 cm, by means of adaptively deforming into a deep arc shape (fig. S26A). Reducing the gap further either causes it to be stuck for extended periods (gap size, 6 cm; fig. S26B) or results in self-snap to move away from the gap (gap size, 4 cm; fig. S26C). Similar pass-through and snap-back behavior is observed with wooden plate walls, albeit with slightly larger minimum gap sizes due to higher friction (fig. S27). In the second case, we introduce a narrow gap to the confined space between two parallel walls (Fig. 9B and fig. S28), where the gap size is 7.5 cm and the spacing between the walls is 10 cm. The robot is initially placed in parallel to the walls and away to the left of the gap. It approaches the gap with zigzag reflections after several snaps and active turns (Fig. 9B). Notably, it can only pass through and escape from the confined space when less than half of its body length is blocked by the wall (fig. S28C, 4.9 to 6 min); otherwise, it snaps back (fig. S28C, 2.9 to 4 min).

Next, we explore its escape performance from mazes with narrow gaps. Ideally, the most challenging scenario will be a maze with narrow gaps in both the dividing walls and the exit. For simplicity, we decompose this scenario into two proof-of-concept demos: one involving a simple enclosed rectangular walled space with a narrow exit gap (~28.6% smaller than the body length; Fig. 9C) and the other featuring a multichannel maze with the same-sized narrow gaps between the walls but a wide exit (Fig. 9D). The soft robot is randomly dropped away from the exit and attempts to escape three times. In the first demo, it must rely on snapping and active turns to precisely locate and reach the exit (i.e., with more than half of the body facing the exit), requiring a considerable amount of time (1 hour and 25 min) and following complex trajectories (Fig. 9E) due to the heightened difficulty (movie S10). In the second demo, the maze consists of two panes separated by a narrow gap, with further divisions through horizontal walls. Notably, locating the gap proves challenging for the soft robot, navigating through it only a few times (once for locations 1 and 2 and three times for location 3; Fig. 9F and fig. S29). The duration for successful escapes varies between ~30 and ~100 min (Fig. 9F, fig. S29, and movie S10).

**Fig. 10.**
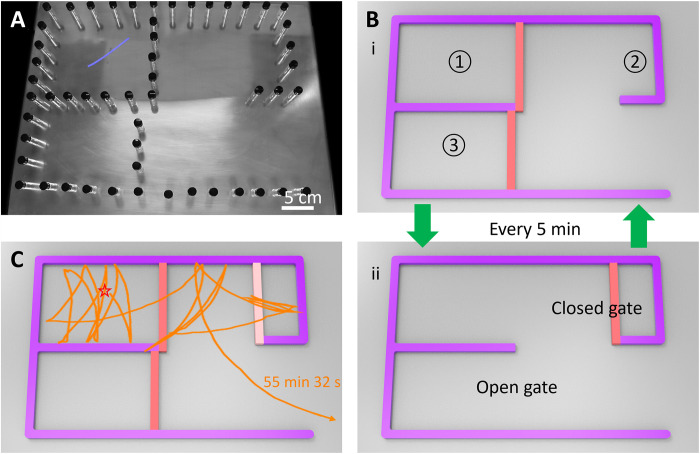
Self-escaping of the hybrid ribbon from a maze with in situ changing pattern. (**A**) The image of the initial pattern of the maze. (**B**) The maze changes the patterns between (i) and (ii) every 5 min. In (i), the gates (orange bar) are closed in areas ① and ③ and open in area ②. In (ii), the gate is open in areas ① and ③ and closed in area ②. (**C**) The corresponding escaping trajectories from random starting points (denoted as star symbols) in area ①. The hot surface temperature is 120°C.

Last, to further demonstrate the escaping versatility of the hybrid ribbon–based soft robot, we introduce a dynamic maze featuring in situ changing patterns that switch between two different layouts every 5 min. Figure 10 (A and Bi) shows the initial maze layout, where areas ① and ③ start as closed areas with closed gates, while area ② has an open gate. After 5 min, it transitions to a different layout depicted in Fig. 10Bii, where areas ① and ③ become open areas with the gates opened, while area ② turns into a closed area. The maze pattern dynamically changes every 5 min, with the gates periodically opening and closing, regardless of the robot’s instantaneous location. As shown in Fig. 10C and movie S11, the robot is randomly placed in the initially closed area ①. Distinct from previous static mazes, this dynamic maze challenges the soft robot not only to find the correct pathway but also to time its movements accurately, introducing additional difficulties to the escaping process. Despite occasional entrapment in the temporarily closed areas, the soft robot successfully escapes from the maze with an in situ changing pattern, showcasing its notable maze-escaping capabilities.

## DISCUSSION

We designed and fabricated a self-navigational hybrid twisted-helical LCE ribbon–based soft robot. It is capable of autonomously maneuvering through complex unstructured confined environments, such as multichannel mazes, without the need for human interventions or external controls. The hybrid ribbon exhibits superior sustained active self-turning capabilities compared to its two twisted and helical counterparts, which can only self-turn transiently. The geometric asymmetry at both ends of the hybrid ribbon accounts for its built-in sustained self-turning, while its counterparts’ nonsustained self-turning results from transient untwisting dependent on handedness. Encoded with PI such as multicapabilities of active self-turning, and self-snapping for reverting self-rolling directions or passive turning when encountering obstacles, the hybrid ribbon–based soft robot can intelligently self-escape from simple and complex mazes with multichannel confined spaces with complex curved zigzag paths on both rigid continuous surfaces and granular surfaces without any human interventions, whereas its counterparts are stuck inside because of the lack of sustained self-turning capabilities. Self-navigation through mazes with narrow gaps and in situ dynamic changing layouts further demonstrates its strong self-adaptivity to more challenging confined and changing environments. This work will be enlightening for soft robotic applications in self-powered autonomous and intelligent open-task navigation and environmental monitoring in unstructured environments. We envision that such active self-turning strategies can be applied to other stimuli-responsive soft materials with different structures and geometries for self-navigation in complex confined unstructured environments.

This work fills the important knowledge gaps and address the limitations of our previous study on twisted LCE ribbon–based autonomous soft robots (*48*). First, the untwisting-induced transient self-turning behavior in twisted LCE ribbons is uncovered in this work. Second, relying on passive self-turning around obstacles and self-snapping in our previous work make it only capable of self-escaping from simple maze-like obstacle courses without confined channels (*48*). This work shows that the lack of active and sustained self-turning in the twisted ribbon makes self-snapping become an adverse effect in self-escaping from parallel spaces, as it leads to the trapping. In addition, it sacrifices self-snapping capability when self-rolling on sand because of the sharp edges in the twisted ribbon. Third, by breaking the geometric symmetry in the twisted ribbon (*48*), the hybrid twisted-helical LCE ribbon in this work gains multicapabilities, including notable active and sustained built-in self-turning, passive turning around obstacles, and enhanced self-snapping, especially in challenging granular terrain. These embodied multicapabilities enable its unprecedented self-navigation abilities in handling substantially more complex and challenging multichannel mazes with more than 20 potential trapping zones than the studied simple maze-like obstacle course without confined channels (*48*). Furthermore, both self-turning and self-snapping behaviors in the new design are well explored and uncovered fundamentally and experimentally. Furthermore, this work showcases the power of combining the soft body compliance and the multicapabilities of active self-turning and self-snapping in handling more challenging mazes that have either narrow gaps or in situ changing layouts, a substantial challenge that rigid robots cannot deal with because of their rigid body shape and lack of active environment adaptivity.

However, there are some limitations to this work. Despite a number of potential trapping subspaces in the designed complex mazes, their complexities are still less than that of regular labyrinth mazes with more intricate pathways and walls. Expanding the maze complexity will require adjustments to the experimental setups, such as increasing hot plate sizes or modifying the sample’s structural design (e.g., a tapered shape). In addition, exploring other shaped mazes, such as circular mazes or mazes with irregular-shaped channels, may yield different self-navigational behaviors. Last, developing a more sophisticated model will enhance our understanding of the dynamics involved in escaping for the samples with built-in geometry asymmetries. These limitations provide avenues for future research and improvements.

## MATERIALS AND METHODS

### LCE sample fabrication

The LCE samples were synthesized by modifying previous reported thiol-acrylate Michael addition reaction method (*49*). The liquid crystal mesogenic monomer 1,4-bis-[4-(3-acryloyloxypropyloxy)benzoyloxy]-2-methylbenzene (RM 257) was purchased from Wilshire Technologies. The chain extender 2,2′-(ethylenedioxy) diethanethiol (EDDET), cross-linker pentaerythritol tetrakis (3-mercaptopropionate) (PETMP), photoinitiator (2-hydroxyethoxy)-2-methylpropiophenone (HHMP), and catalyst dipropyl amine (DPA) were purchased from Sigma-Aldrich. In a typical synthesis process, 2 g of RM 257, 0.42 g of EDDET, 0.18 g of PETMP, and 0.012 g of HHMP were dissolved in 0.7 g of toluene at 85°C with magnetic stirring. Then, 0.29 g of 2% DPA solution was added at room temperature. After being fully mixed and degassed, the solution was carefully poured in the pre-prepared molds. The samples were placed in a closed container for 1 day for full reaction, followed by drying in an oven at 70°C for 1 day. Next, the samples were uniaxially stretched to ~100% strain and twisted while exposed to 365-nm UV irradiation at 20 mJ/cm^2^ for 10 min.

### Characterization methods

The high-speed videos were taken with a high-speed camera (Photron FASTCAM SA3) with a frame rate 125 fps. The maze escaping was performed on a hot plate with aluminum surface. For the sandy terrains, the sand bed thickness (>10 mm) is more than three times higher than the sample diameters.

### FEA simulation

FEA simulation was performed with commercial software Abaqus/Explicit. LCE samples were modeled as linear elastic materials with a Young’s modulus of 11 MPa and Poisson’s ratio of 0.3. The simulation methods of twisting/untwisting induced by heating/cooling were discussed in our previous work (*48*). Anisotropic thermal expansion coefficients α*_ij_* were defined in a cylindrical coordinate, while α_33_ denoted the sample length shrinkage and α_23_ was the untwisting coefficient.

The self-turning process in Fig. 5A was simulated with an untwisting process without rolling. Here, the sample was contacting a rigid surface with friction coefficient 0.1. During the untwisting process, the center of the sample was translational constrained but free to rotate. Gravity (1 mN) was applied, and the sample can self-turn during the untwisting because of the friction. The self-turning with rolling process in Fig. 5B was simulated with multistep. First, the sample was placed on a rigid surface with friction coefficient 0.1. Then, the sample was compressed to mimic the bended arc body during the rolling process. Second, the sample was heated to untwist. Simultaneously, rotation was applied on the center of the sample along the sample length to simulate the rolling process. The sample can roll forward because of the friction between the sample and the surface, and the sample can self-turn because of the untwisting process. Third, the sample stopped to be heated, and only the rotation was applied. This was to simulate the rolling process without turning when the sample reached the temperature equilibrium and untwisting stopped. The rolling processes at different temperatures were also performed (fig. S12).

## Supplementary Materials

### This PDF file includes:

Notes S1 to S5 Figs. S1 to S29 Legends for movies S1 to S11
