# Supplementary material for: Physically intelligent autonomous soft robotic maze escaper
Source: Sci Adv. 2023 Sep 8;9(36):eadi3254. doi: 10.1126/sciadv.adi3254 (PMC10491293; doi:10.1126/sciadv.adi3254)
Supplement: Supplementary file 1 — Notes S1 to S5 Figs. S1 to S29 Legends for movies S1 to S11 [file sciadv.adi3254_sm.pdf]

Science Advances
AAAS

Supplementary Materials for

Physically intelligent autonomous soft robotic maze escaper

Yao Zhao *et al.*

Corresponding author: Hao Su, hsu4@ncsu.edu; Jie Yin, jyin8@ncsu.edu

Sci. Adv. **9**, eadi3254 (2023)
DOI: 10.1126/sciadv.adi3254

The PDF file includes:

Notes S1 to S5
Figs. S1 to S29
Legends for movies S1 to S11

Other Supplementary Material for this manuscript includes the following:

Movies S1 to S11

Supplementary Text

Note S1: Discussion on the twisted ribbon and helical ribbon

The twisted ribbon in this manuscript refers to the drill bits-like ribbon. As shown in **fig. S2A**, the twisted ribbon has a straight centerline that also acts as the centerline for the helix edges. Nonetheless, the hierarchical twisted helical ribbon in the manuscript differs from the twist-free helical structure. As shown in **fig. S2B**, the cross-section centers of the twist-free helical structure do not align a straight line. Instead, the ribbon coils around the helical centerline. The hierarchical twisted helical ribbon exhibits a two-order hierarchical structure that contains higher-order helical waves and lower-order twists (**fig. S2C**).

Fig. S2D illustrates the helical sample fabrication method. First, an LCE ring with 5 mm in radius is obtained by a mold after the 1st stage curing (**fig. S2E**). Then, the ring is cut and stretched, and a helix shape can be formed even without twisting (**fig. S2F**).

Fig. S2G shows a twist-free helical ribbon. It should be noted that a twist-free sample cannot maintain continuously self-rolling on hot surfaces.

Note S2: Discussion on the temperature gradient along the sample widths

Fig. S6 shows that all the samples have a temperature gradient along the sample widths with ~ 15 ^{o}C drop from the contacting regions to the outer boundary. The IR images were taken with an infrared camera (FLIR SAC300). As the samples roll forward, the previous contacting areas will detach from the hot plate and start to be cooled by the ambient air, while the newly contacted areas will be heated by the hot plate. In this way, the dynamic heating and cooling process enables the samples to reach a temperature gradient equilibrium which acts as the driving force for the continuous self-rolling.

Note S3: Discussion on the hybrid sample turning capabilities with different helical-twisted ratios

During rolling, the helical and the twisted region share the same rolling angle θ (**Fig. S15**). The velocity at the intersecting point of the two regions is given by

$$v_{in} = \frac{c(d_h\theta - d_t\theta)}{2(1+c)} + d_t\theta/2 = \frac{c(d_h\theta - d_t\theta)}{2(1+c)}$$

where d_h and d_t denote the diameter of helical and twisted region, respectively. Notably, d_h and d_t increase with temperature increasing. c is the ratio of the helical-region length and the twisted region length. The angular velocity of self-turning is given by

$$w = \frac{d_h\theta - d_t\theta}{l}$$

where l is the length of the escaper that is temperature dependent. Thus, considering the cancelation from the scattering in the velocity of line elements of the escaper, we arrive at the correlation between the ratio $d\varphi/ dX$ and c

$$\frac{d\varphi}{dX} = \frac{\sigma w}{\sigma_{max} v_{in}} = \frac{2\sigma(d_h - d_t)(1+c)}{l\sigma_{max}(cd_h + d_t)}$$

where σ *is* standard deviation in the velocity of the line elements of the escaper and is a function of c. $\sigma_{\max}$ denotes the maximum deviation in the velocity of the elements when c =1.

Note S4: Discussion on the snap-through process on rigid surface and sand

A self-snap-through occurs if the middle parts of the samples are blocked by obstacles (**figs. S16, S17,** and movie S4). The three samples have similar behaviors on rigid hot surfaces. Specifically, the shallow arc shapes are flattened by the samples, trying to align with the obstacles from the top view (**figs. S16**). When forming the unstable flattened shapes, the samples also generate out-of-plane buckling from the side view (**fig. S17A-C**). The samples then snap-through via a sudden falling away from the obstacle, by which the rolling direction flips.

We then explore the snap-through processes on sand. As expected, the snap-through becomes harder on sand comparing to on rigid surfaces (movie S5). The snap time of the helical and hybrid samples increase from ~100 ms and ~100 ms to ~150 ms and ~250 ms, respectively (**Figs. 7C, D**). Nonetheless, the twisted sample is stuck and cannot snap on sand (**Fig. 7B**). The main reason is that the samples can burrow into the sands and pile two little sand dunes in the vicinities of the two ends, which significantly increase the snap difficulties (**fig. S17D**). In contrast, the helix shapes in the helical and hybrid samples can hinder the burrowing and exhibit better snapping performances than the twisted sample (**Fig. 7B-7D, fig. S17E-F**).

Note S5: Discussions on the hybrid sample dimensions

To study the influences of sample dimensions, two groups of hybrid samples have been fabricated. The first group of samples have the same square cross-sections with side length a (i.e., 2.5 mm) but different lengths l (i.e., 5 cm, 7 cm, 10.5 cm, and 12 cm). The second group of samples have the same lengths l (i.e., 10.5 cm) with different side length a (i.e., 1 mm, 2.5 mm, and 5 mm).

Generally, most of the hybrid samples with different dimensions can free roll, make active and passive turnings, and snap, which are similar to the hybrid sample in the manuscript. However, each sample behaves differently in terms of the turning and snapping temperatures. For example, as shown in **fig. S19 A** and **B**, the sample with a = 2.5 mm and l = 5 cm tends to snap rather than passive turn even if the sample end is blocked by an obstacle when placed on a hot surface with 90 ºC. On the contrary, the sample with a = 2.5 mm and l = 12 cm tends to passive turn when a sample end is blocked by an obstacle. The reason is that the shorter length will result in smaller rolling driving force F and smaller distance b between the contacting point and the sample center, thereby generating a smaller turning moment. If the turning moment is too small to overcome the reaction moment induced by friction, the sample will tend to snap (**fig. S19A**). Conversely, the sample will tend to make passive turns (**fig. S19B**), if the turning moment is large enough. In this way, it is easier for the shorter sample to snap while it is easier for the longer sample to make passive turns. Furthermore, we speculate that if the aspect ratio is infinitely large, the sample may not be able to snap due to its extremely low rigidity. In such cases, the sample can only be considered as a thread rather than a ribbon.

We further studied the influences of a. It is found that the sample with a = 5 mm and l = 10.5 cm also tends to snap when the sample end is blocked by an obstacle, as shown in **fig. S19C**. This behavior can be attributed to the fact that the specific driving force F (driving force by weight) is too small to overcome the frictions for turnings. The specific driving force F can be estimated with the sample rolling speed. For example, the rolling speed of the sample with a = 5 mm and l = 10.5 cm significantly dropped to ~ 1.8 mm/s on 120 ºC hot surface while the rolling speed of the sample with a = 2.5 mm and l = 10.5 cm is ~ 3.1 mm/s. The reason is that the larger diameter will lead to lower temperature at the core of the ribbon, and the core will turn into a pure payload instead of providing driving force. Hereby, we speculate that the ribbon will stop free rolling if a is infinitely large.

We also found that the sample with a small a (e.g., a = 1 mm and l = 10.5 cm) cannot sustain self-rolling on hot surface from 50 to 220 ºC. As shown in **fig. S19D**, the sample can only roll for ~ 6 s and then stopped rolling. The reason is that the small driving force of the sample with small a makes the sample highly sensitive to defects. If the sample is stuck by any defects during rolling, the sample will be quickly heated up and lose the thermal gradient on the sample width, thereby disrupting its rolling motion.

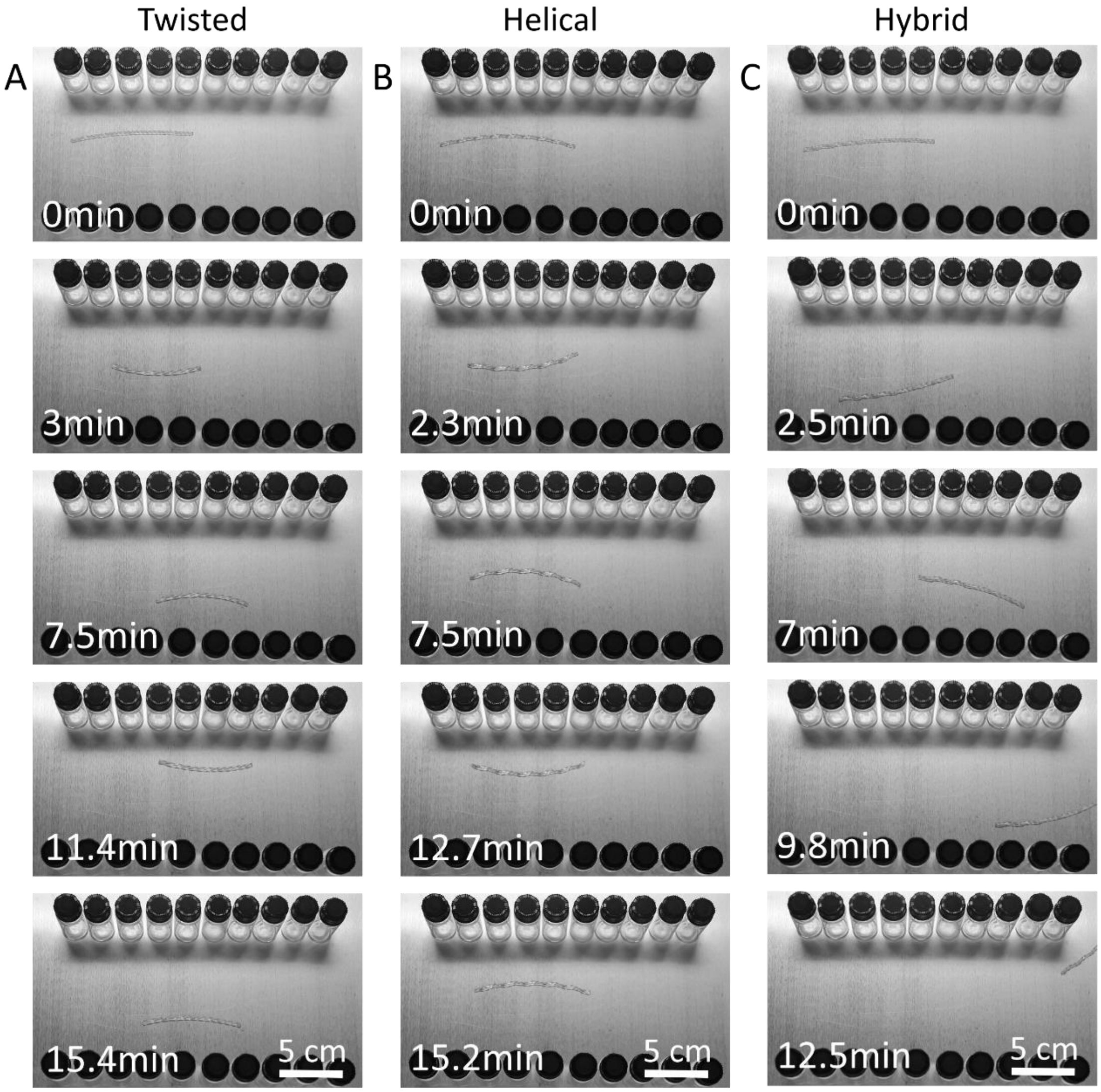

Figure S1. The time lapses of samples escaping performances in the parallel confined space when they are initially parallel to the walls. The twisted **(A)** and helical **(B)** samples are trapped the confined space. The hybrid sample **(C)** can escape from it. The surface temperature is 120 ^{o}C.

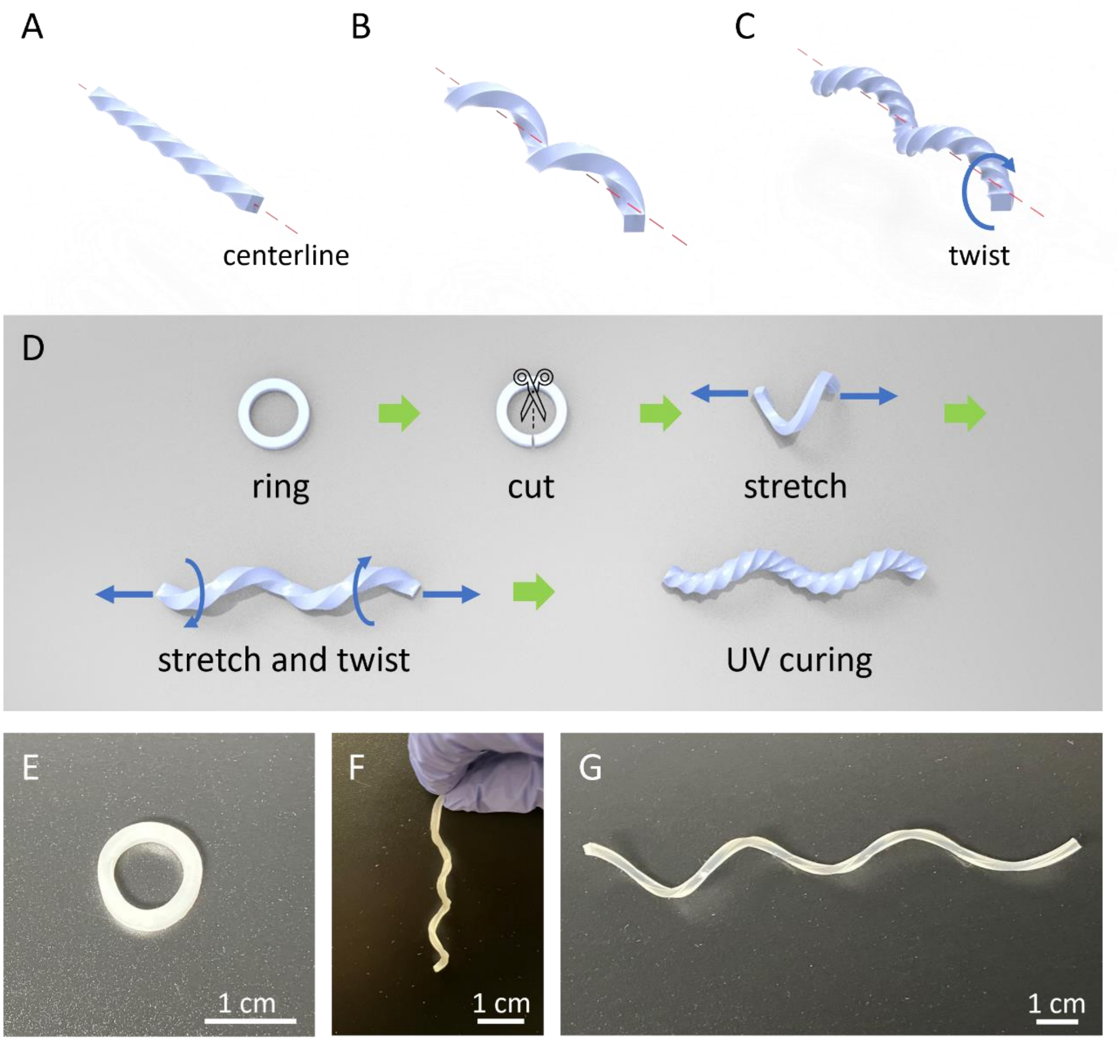

Figure S2. The fabrication method of the helical sample. The schematics of a twisted ribbon **(A)**, a twist-free helical ribbon **(B)**, and a hierarchical twisted helical ribbon **(C)**. **(D)** The fabrication method for the hierarchical twisted helical ribbon. **(E)** A LCE ring after the 1st stage curing. **(F)** A helical shape can be formed if the ring is cut and stretched. **(G)** A twist-free helical ribbon after UV curing.

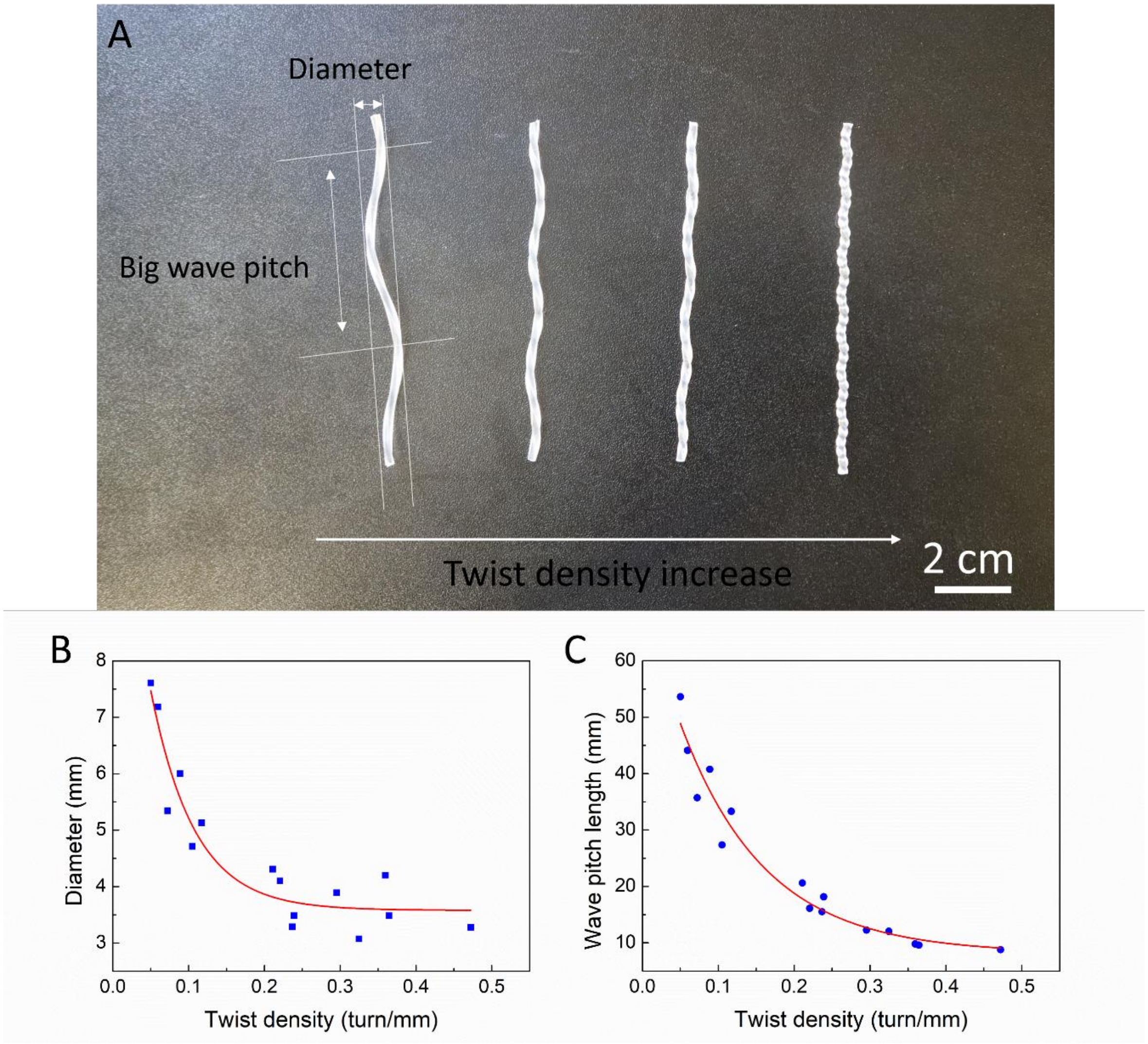

Figure S3. The wave pitch lengths and diameters of the helical samples decrease with higher twist density. (A) The image of samples with different twist densities. The diagrams of the sample diameter **(B)** and wave pitch length **(C)** with twist densities.

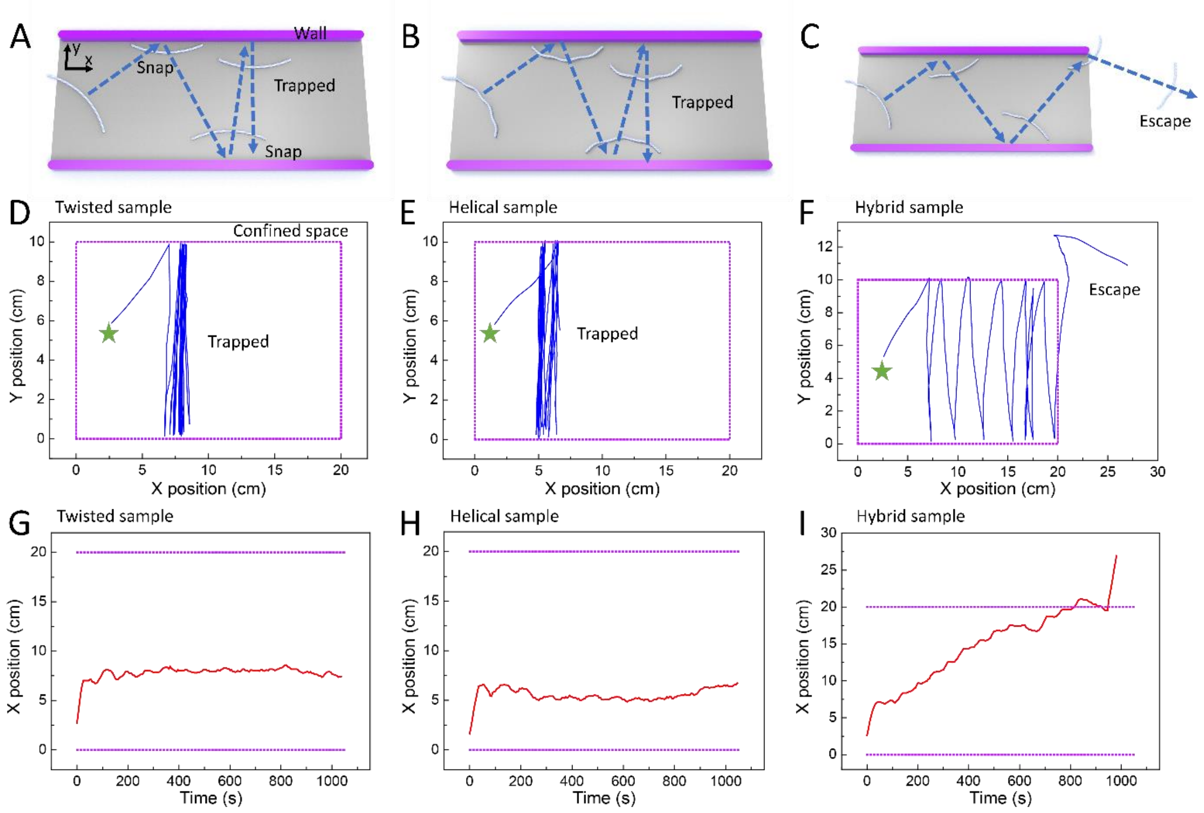

Figure S4. The escaping performances of the three samples from the parallel confined space starting with samples facing the wall. The twisted **(A)**, helical **(B)**, and hybrid **(C)** samples are placed at the same locations with the same angles in the parallel confined space initially. The surface temperature is 120 ^{o}C. The trajectories **(D-F)** and the X positions with time **(G-I)** of the twisted, helical, and hybrid samples, respectively. The confined space is denoted with purple boxes in the figure.

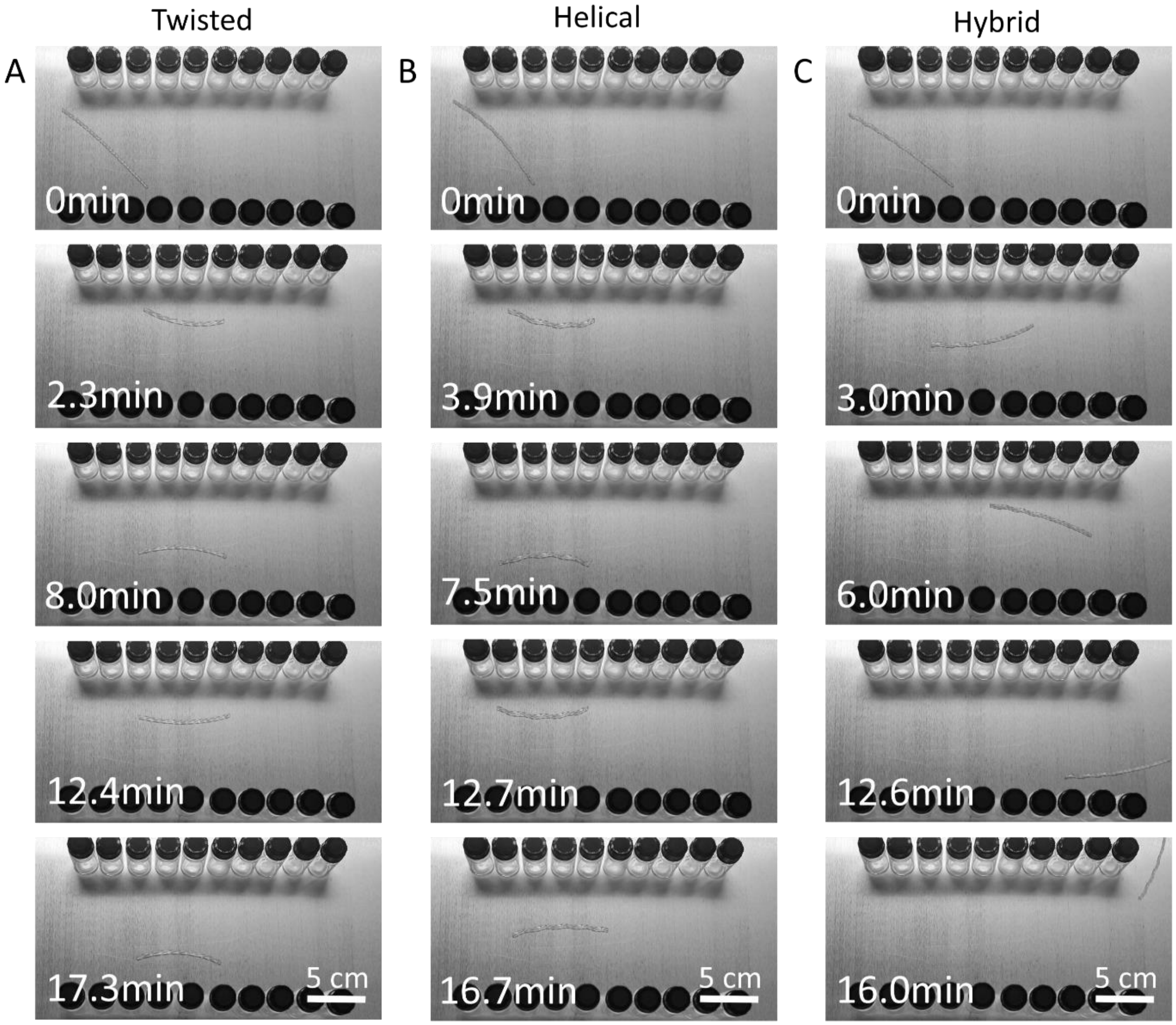

Figure S5. The time lapses of samples escaping performances in the parallel confined space when they start with the same angle facing to the wall. The twisted **(A)** and helical **(B)** samples are trapped the confined space. The hybrid sample **(C)** can escape from it. The surface temperature is 120 ^{o}C.

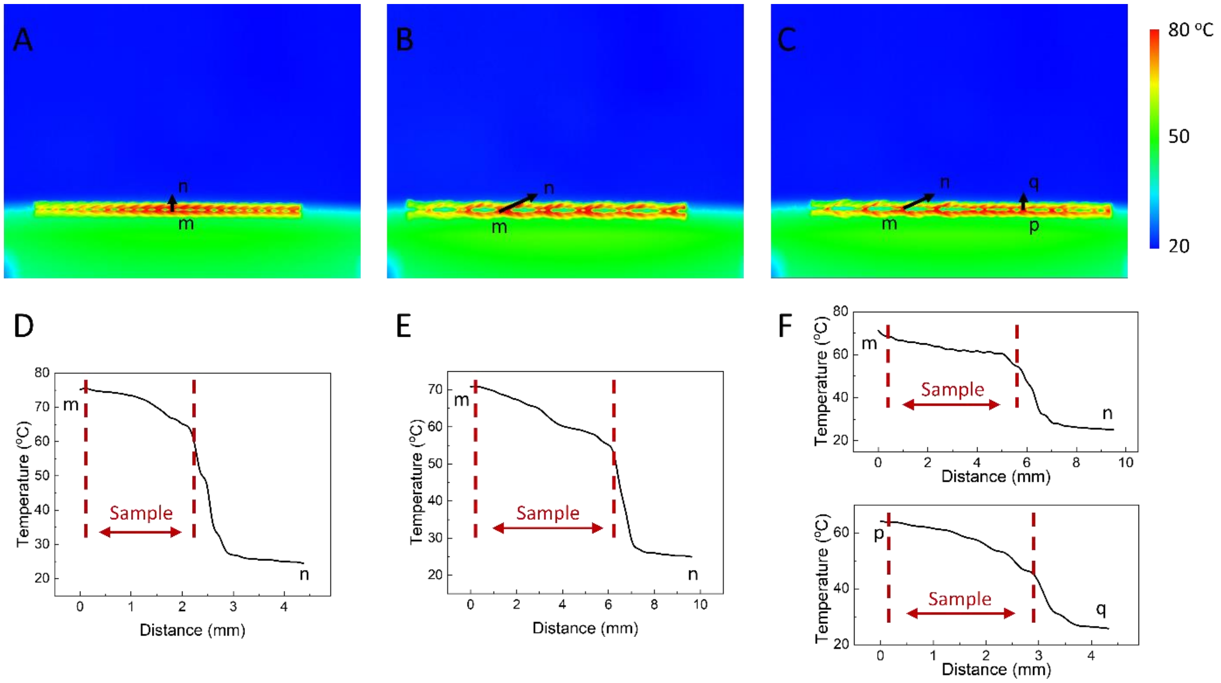

Figure S6. The thermal gradients of the samples. The infrared images of the twisted **(A)**, helical **(B)**, and hybrid **(C)** samples rolling on a 120 °C hot plate. **(D-F)** The temperature profiles along the path mn (and pq in F) in A-C.

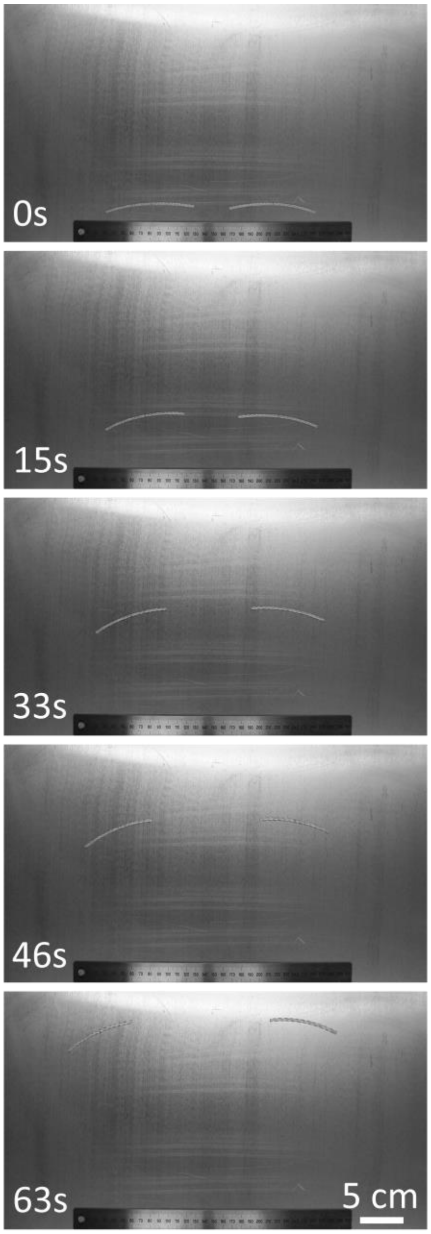

Figure S7. The time-lapses of twisted sample with opposite handednesses. The surface temperature in this figure is 120 ^{o}C.

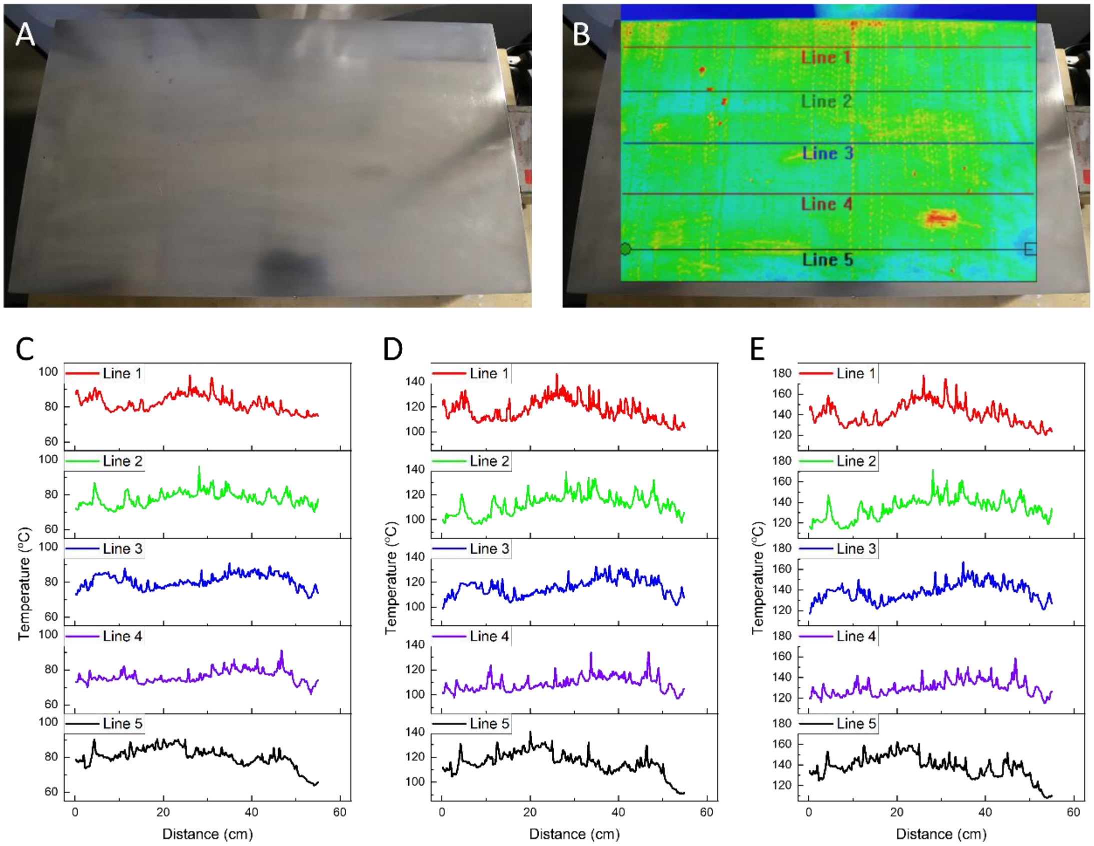

Figure S8. The temperature distributions of the hot plate. (A) The image of the hot plate. **(B)** The infrared image of the hot plate with 80 °C. **(C-E)** The temperature profiles of the hot plate at 80, 120, and 140 °C, respectively.

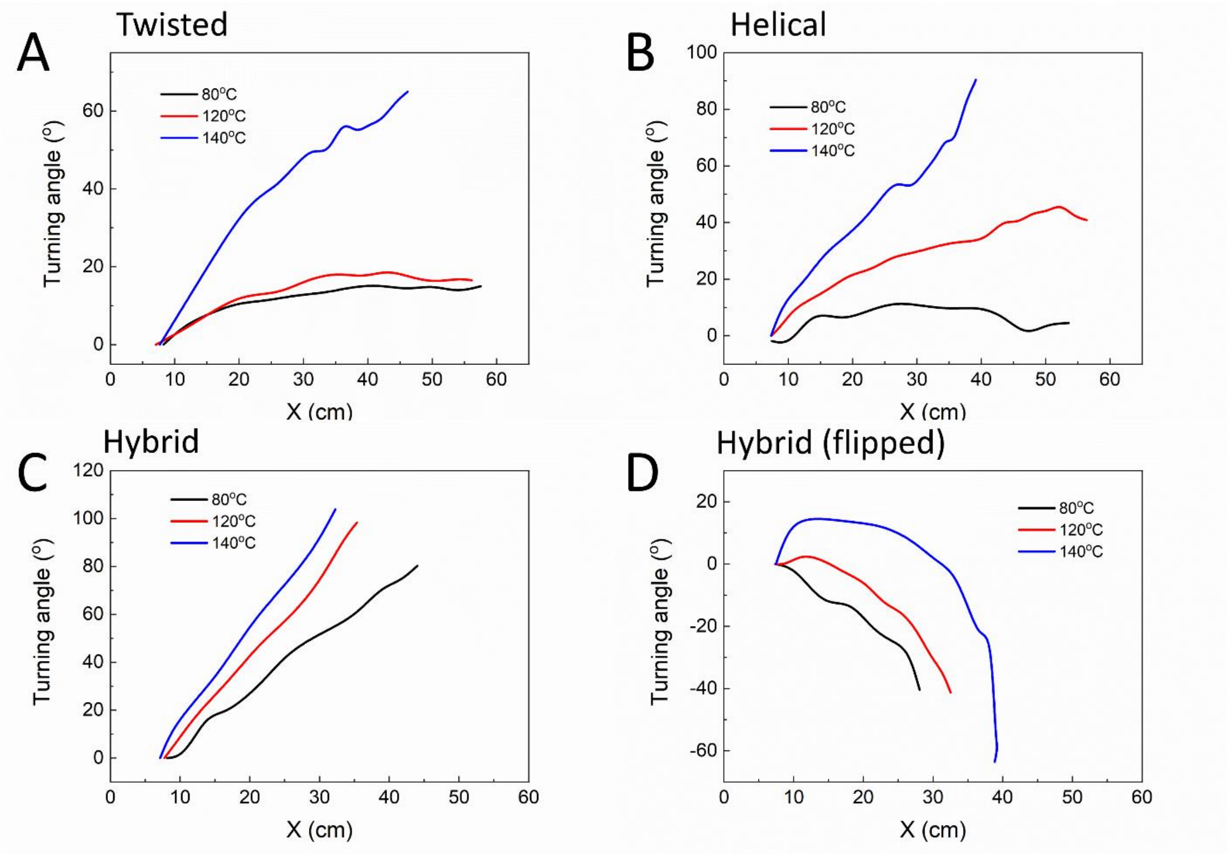

Figure S9. Surface temperature effects on the turning angles. The turning angles of the twisted ribbon **(A)**, helical ribbon **(B)**, and the hybrid ribbon before **(C)** and after flipping **(D)**, which correspond to Figure 4A-B, respectively.

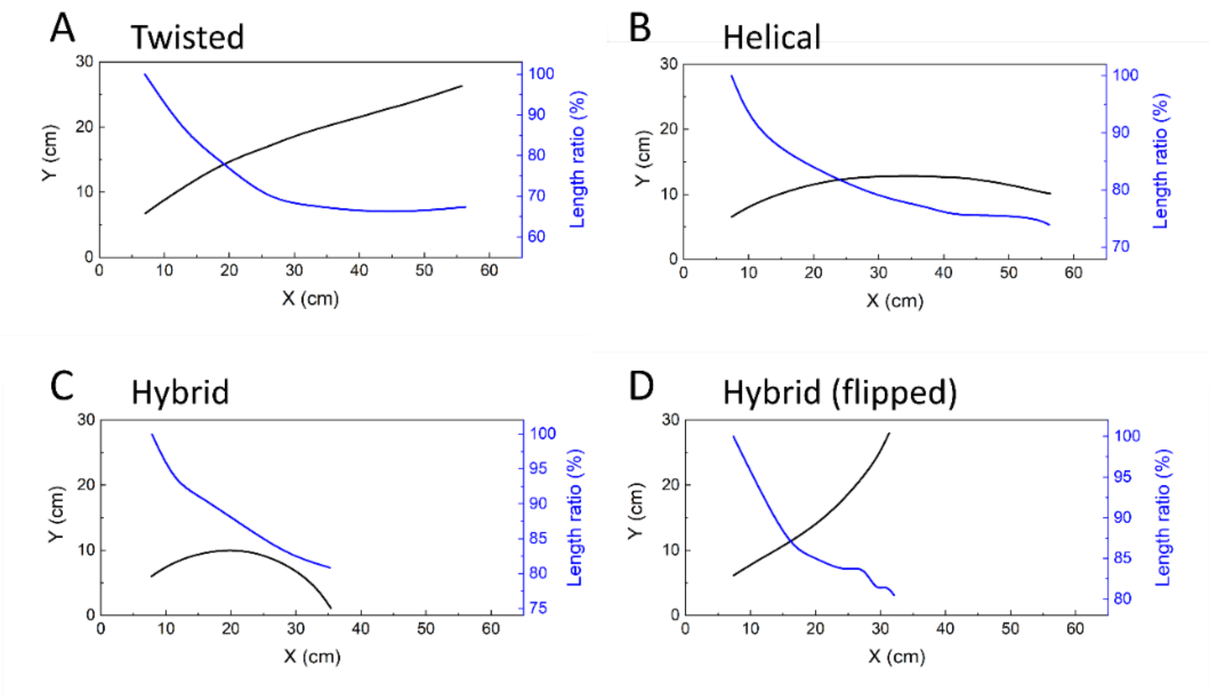

Figure S10. The trajectories and the instant length ratios of the samples. The trajectories and the instant length ratios of the twisted ribbon **(A)**, helical ribbon **(B)**, and the hybrid ribbon before **(C)** and after flipping **(D)**, corresponding Figure 3A-D.

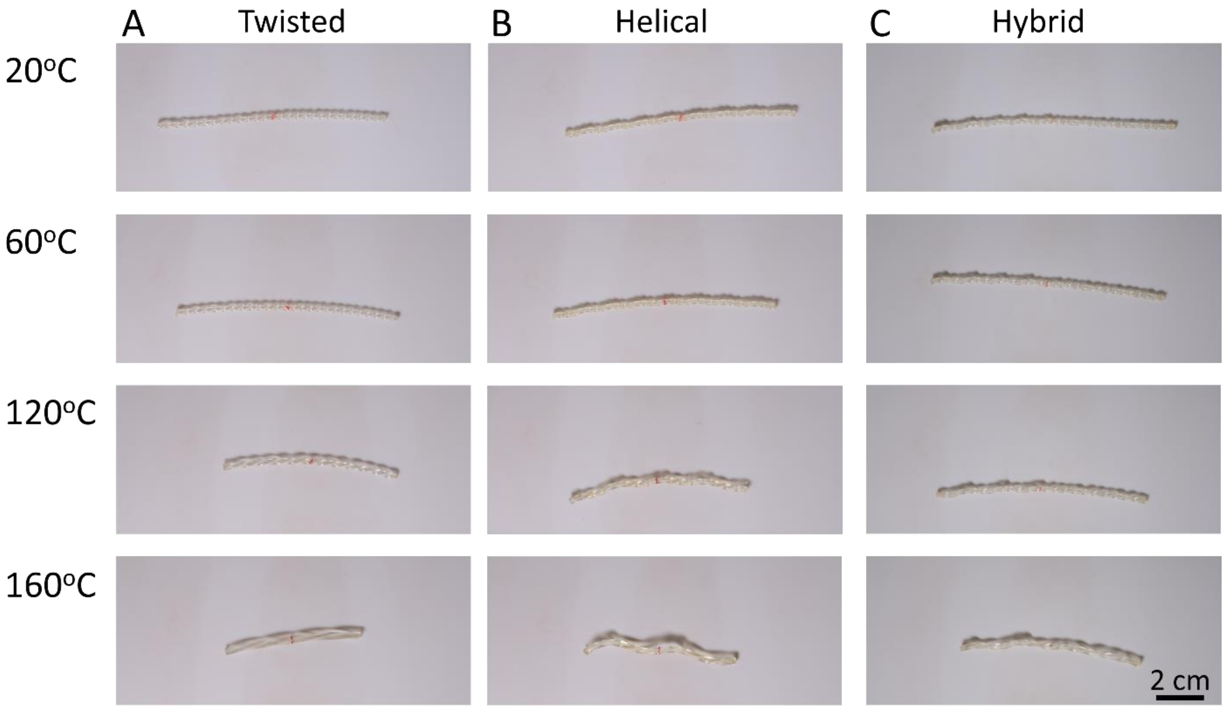

Figure S11. Images of the samples at different surface temperatures. The twisted **(A)**, helical **(B)**, and hybrid sample **(C)** shrunk in length and untwisted at elevated temperatures. The diameters of the helical sample and the helical portion of the hybrid sample also increase at elevated temperatures.

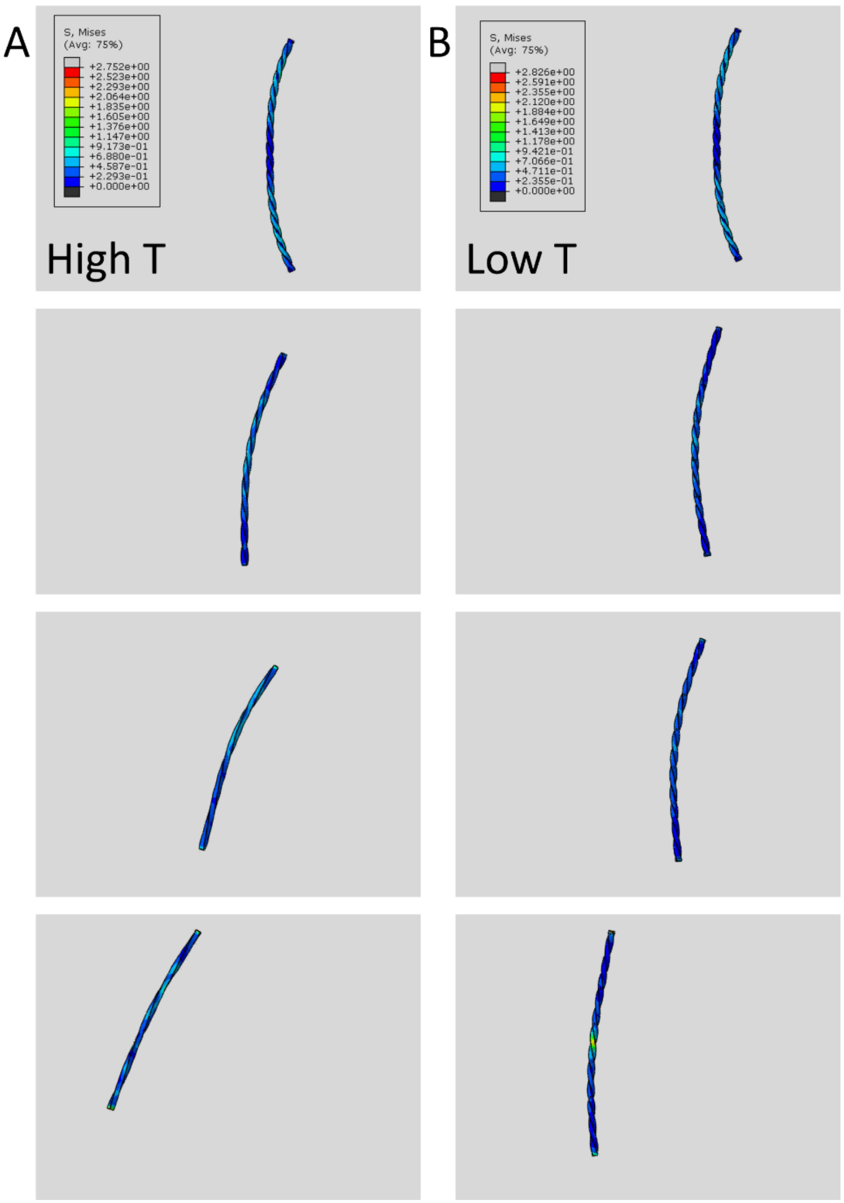

Figure S12. The FEA simulations of the untwisting induced turning. (A) and **(B)** are the turning performance at high and low temperatures, respectively.

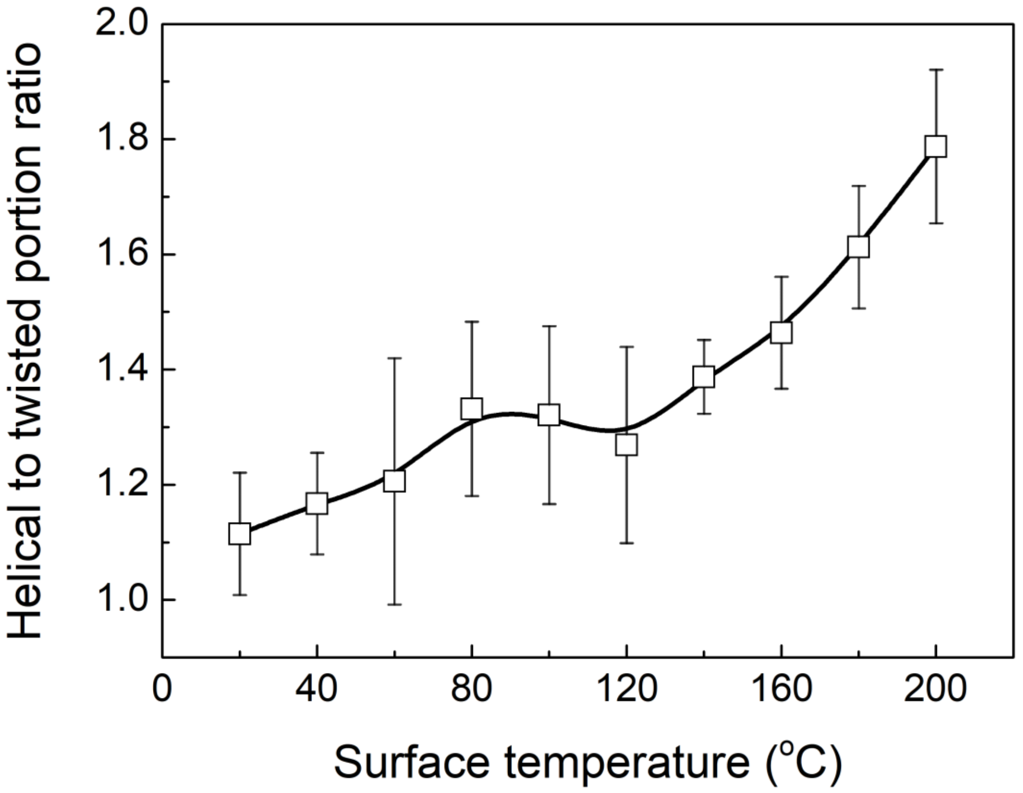

Figure S13. The diameters of the helical portion to the twisted portion ratios in the hybrid sample at different temperatures. The ratio increases with increasing surface temperature.

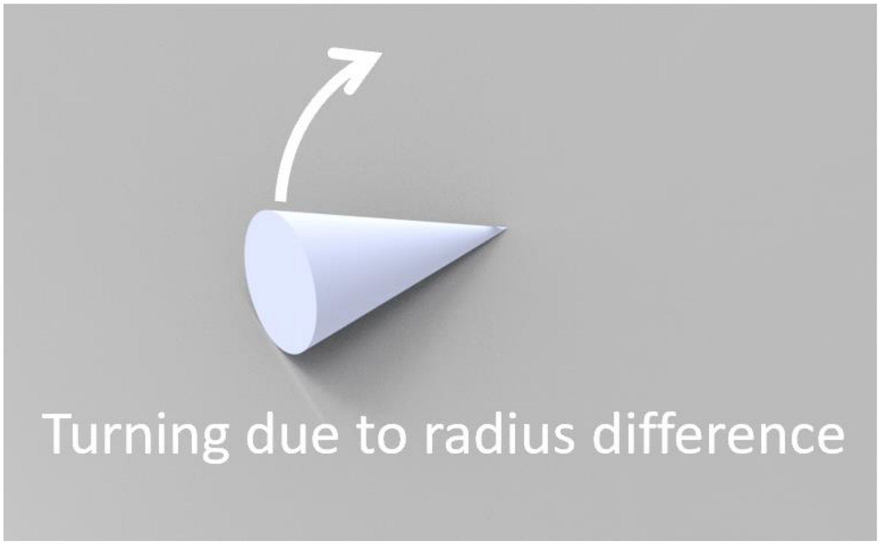

Figure S14. The schematic of a cone making active turning. The geometry asymmetry is attributed to radius difference.

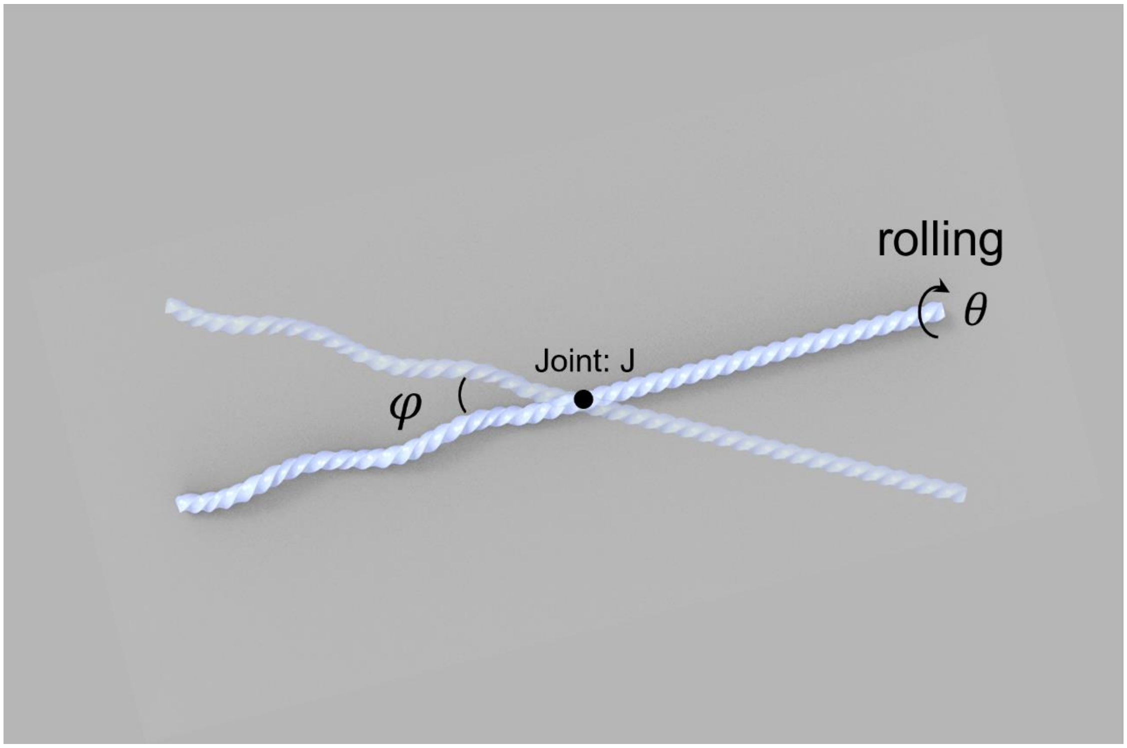

Figure S15. Definitions of parameters during rolling and turning. The turning angle and rolling angle denote as φ and θ, respectively.

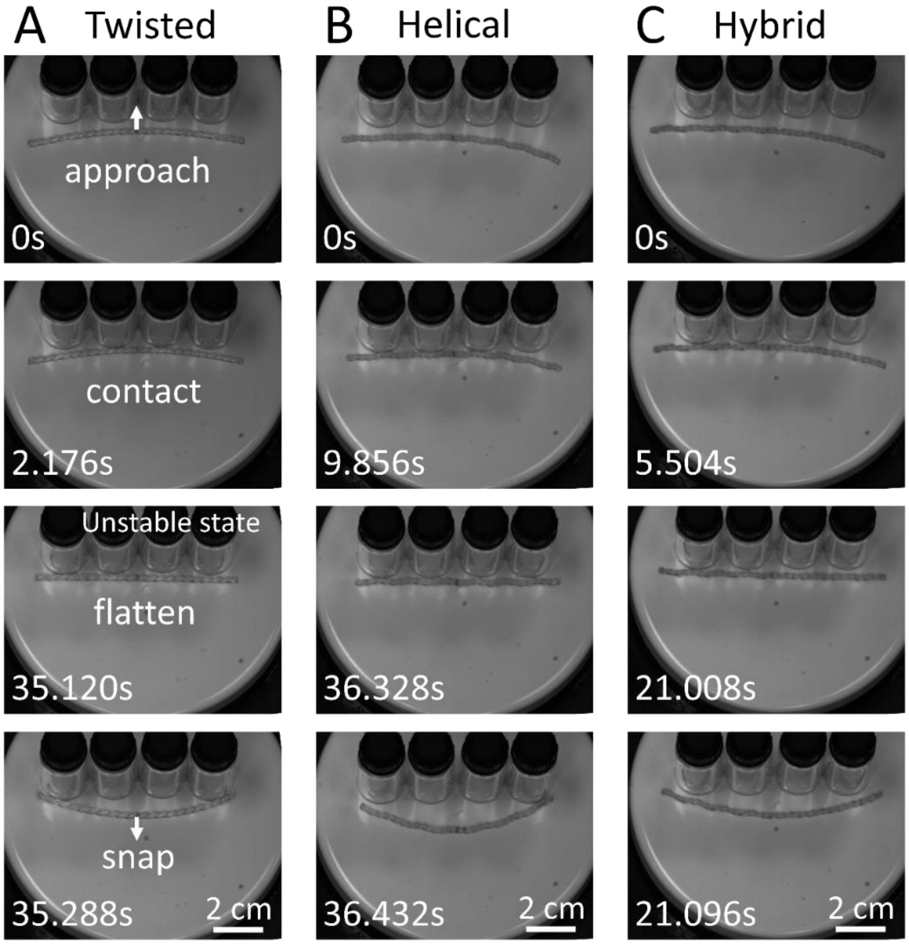

Figure S16. The snap-through processes of samples on rigid surfaces. The high-speed images of the twisted **(A)**, helical **(B)**, and hybrid **(C)** samples during the snap-through processes. The surface temperature is 120 ^{o}C.

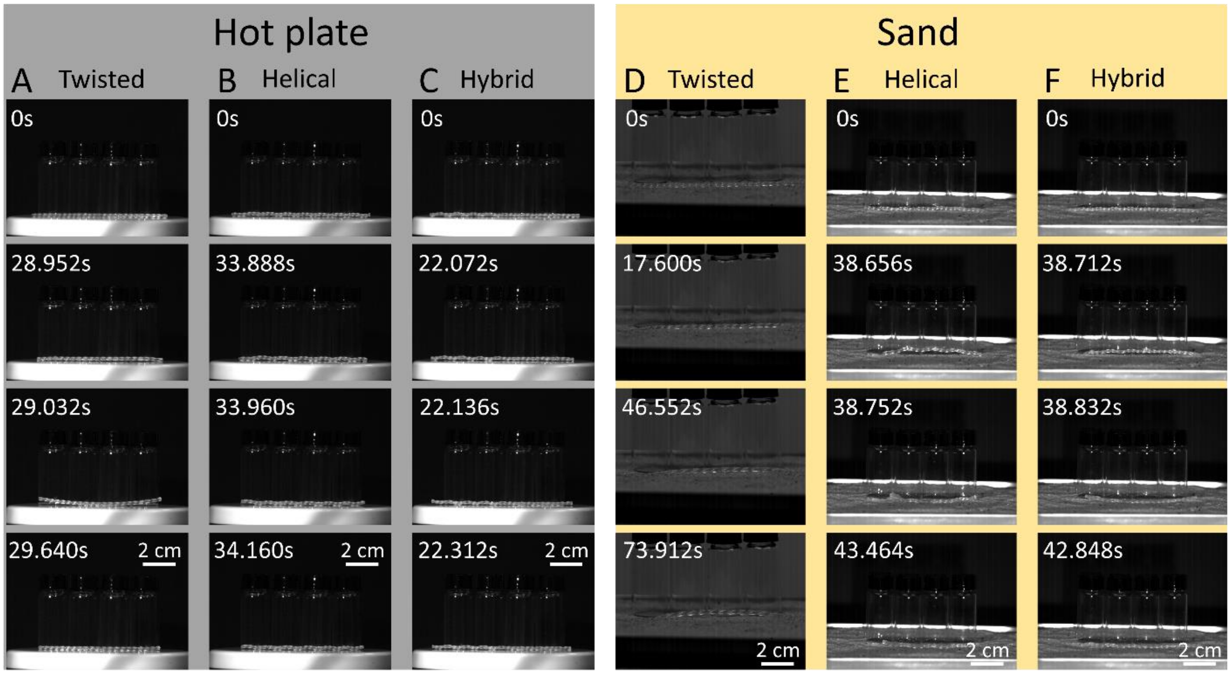

Figure S17. The side view of the snap-through processes. (A-C) The snap-through processes of the twisted **(A)**, helical **(B)**, and hybrid **(C)** samples on rigid surfaces. **(D)** The twisted ribbon is stuck in sands and cannot snap-through. **(E and F)** The snap-through processes of the helical **(E)** and hybrid **(F)** samples on sand. The surface temperature is 120 ^{o}C.

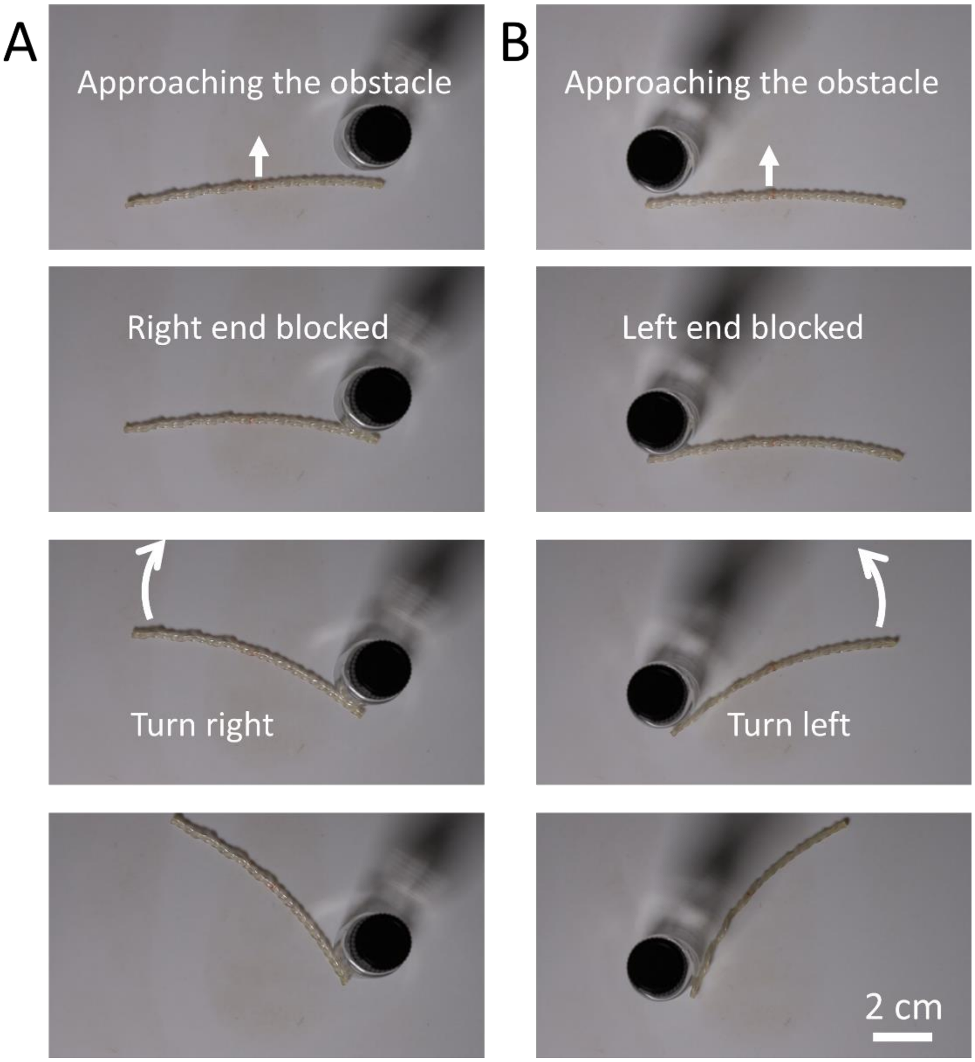

Figure S18. The passive turning of the hybrid ribbon. When one end is blocked by obstacles, the sample can make right turn **(A)** or left turn **(B)** passively.

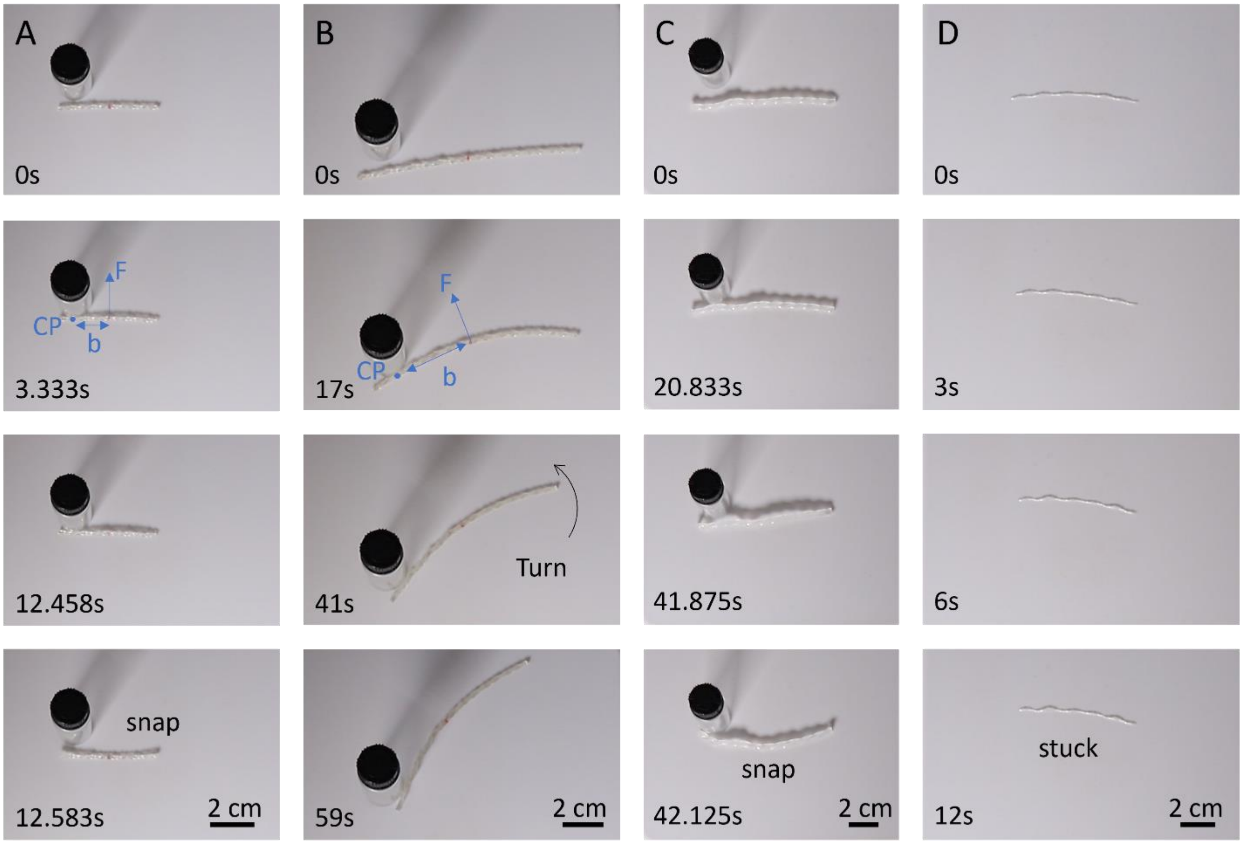

Figure S19. The influences of hybrid sample dimensions. (A) The sample with *a* = 2.5 mm and *l* = 5 cm tends to snap rather than passive turn even if the sample end is blocked by an obstacle. The surface temperature is 90 ^{o}C. (CP: contacting points; F: the rolling driving force; b: the distance between the CP and the sample center). **(B)** The sample with *a* = 2.5 mm and *l* = 12 cm tends to passive turn when a sample end is blocked by an obstacle. The surface temperature is 90 ^{o}C. **(C)** The sample with *a* = 5 mm and *l* = 10.5 cm also tends to snap when the sample end is blocked by an obstacle. The surface temperature is 120 ^{o}C. **(D)** The sample with *a* = 1 mm and *l* = 10.5 cm can only free roll a small distance on 60 ^{o}C hot surfaces.

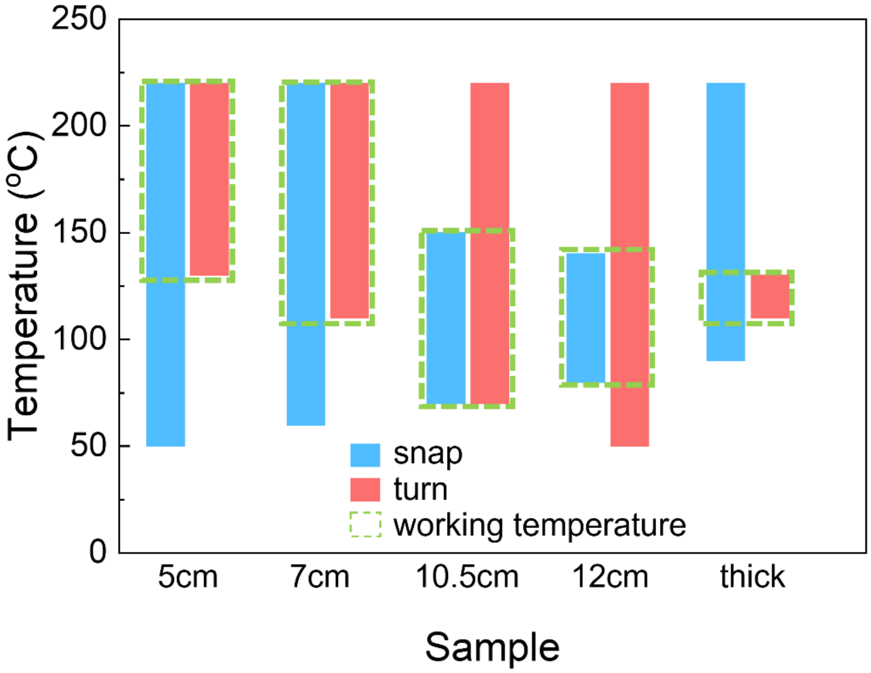

Figure S20. The temperature ranges within which the samples can snap (blue) or make passive turns (red). The 5cm, 7cm, 10.5cm, 12cm samples correspond to the samples with *a* = 2.5 mm and *l* = 5, 7, 10.5, 12 cm, respectively. The thick sample represents the sample with *a* = 5 mm and *l* = 10.5 cm. The overlapping regions (green dashed box) indicate the sample working temperatures for escaping from mazes. The sample with *a* = 1 mm and *l* = 10.5 cm is not listed in this diagram since it cannot sustain self-rolling at this temperature range.

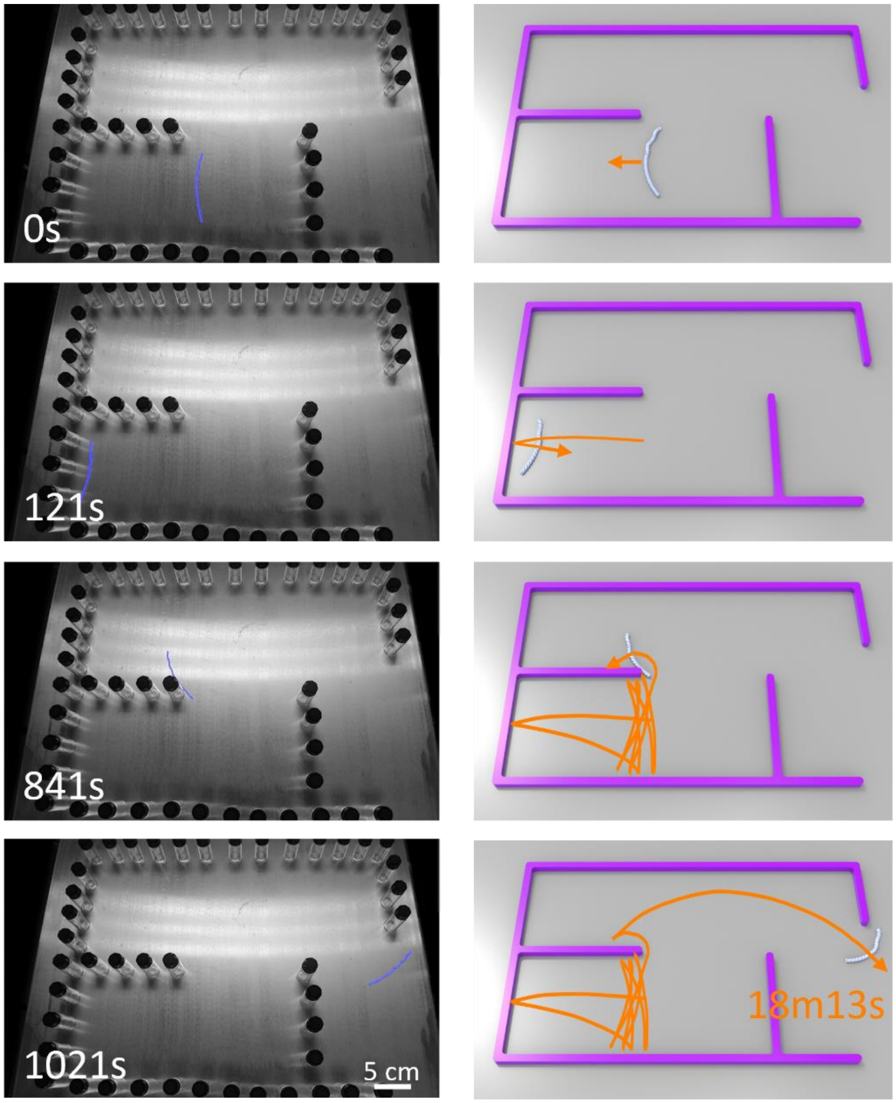

Figure S21. The hybrid sample escapes from Maze 2 in movie S6. The left and right columns are the time-lapse images and schematics with trajectories for the escaping processes, respectively. The surface temperature is 120 ^{o}C.

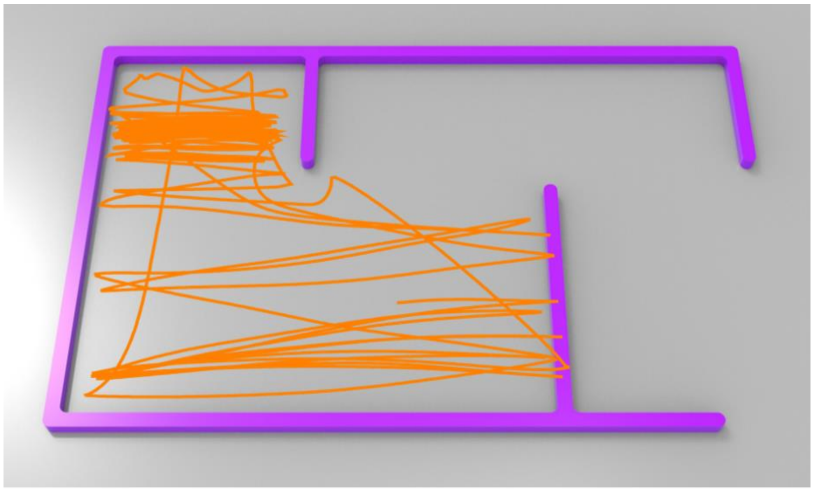

Trapped for 2.12h

Figure S22. The twisted ribbon is trapped in the maze for more than 2 h. The trajectory shows the ribbon is mainly bouncing back and forth between parallel walls. The surface temperature is 120 ^{o}C.

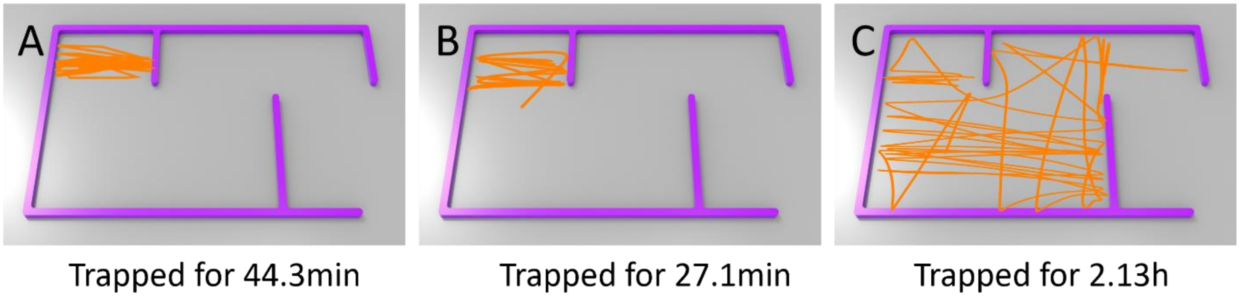

Figure S23. The helical sample is trapped in a simple maze. In movie S7, the helical sample is trapped for 44 min, 27 min and 2.1 h, respectively, starting from Location 1 **(A)**, Location 2 **(B)**, and Location 3 **(C)**. The surface temperature is 120 ^{o}C.

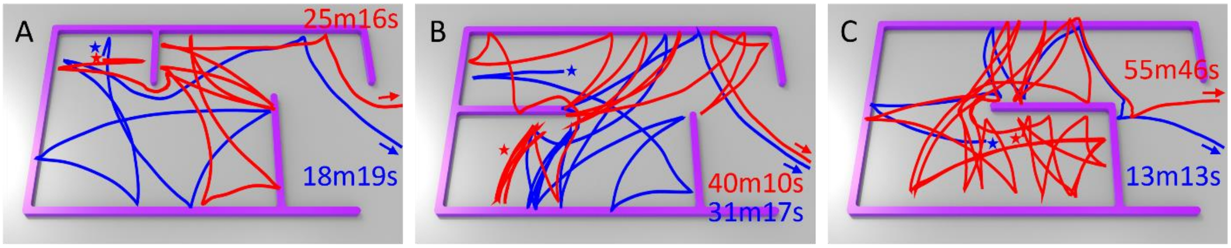

Figure S24. Two other representative escaping paths for the simple mazes. (A-C) correspond to Maze 1, Maze 2, and Maze 3 in movie S6, respectively. The starting points are denoted as star symbols. The surface temperature is 120 ^{o}C.

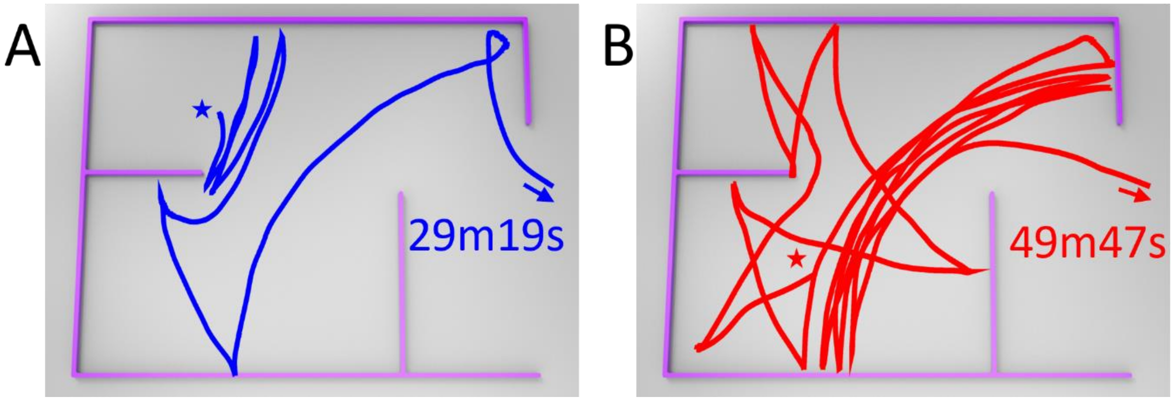

Figure S25. The paths for the hybrid sample escaping the maze on sand. (A) and **(B)** correspond to the cases starting from Location 2 and Location 3 in movie S8, respectively. The starting points are denoted as star symbols. The surface temperature is 120 ^{o}C.

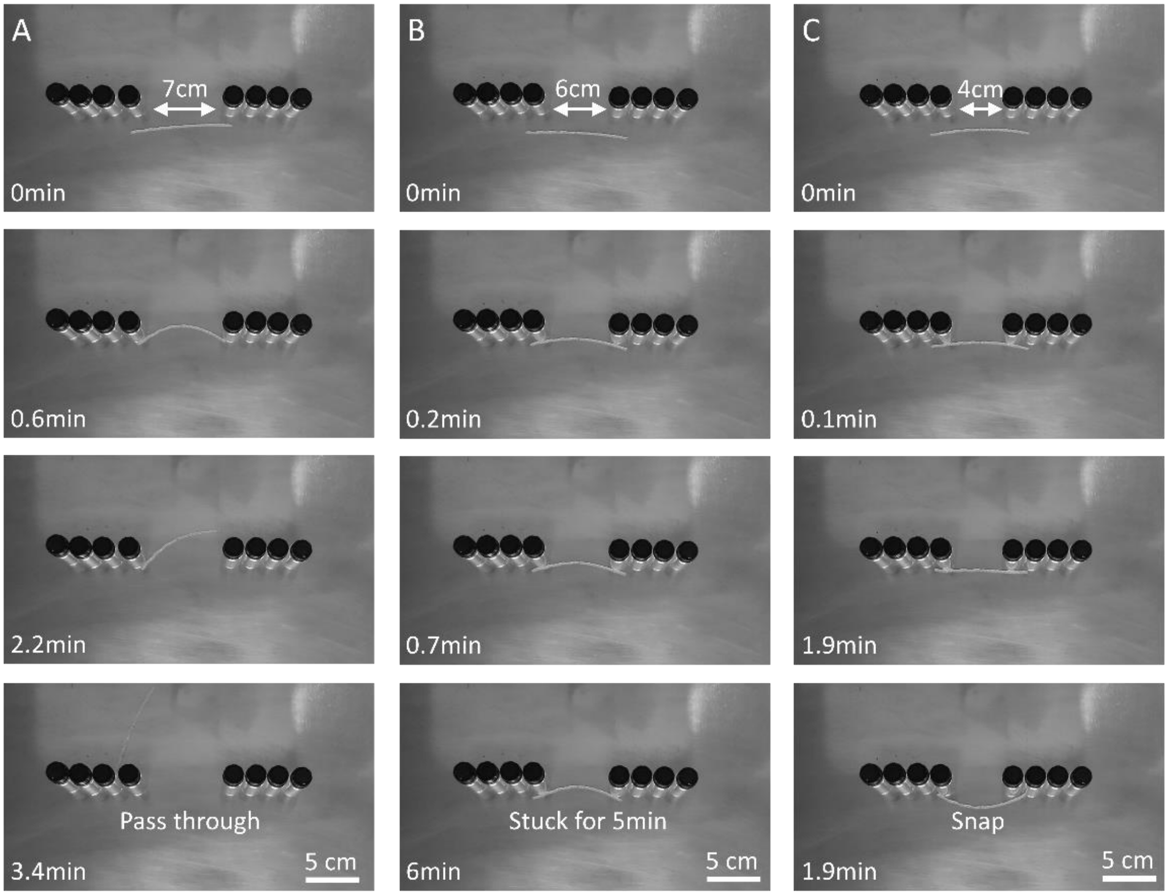

Figure S26. The hybrid sample interacts with narrow gaps constructed with lined glass bottles. (A) The sample can pass through a narrow gap with a width of 7 cm (~ 33.3% smaller than the sample length of 10.5 cm). **(B)** The hybrid sample is stuck for more than 5 min if the gap width is decreased to 6 cm. **(C)** The hybrid sample snaps when encountering a narrow gap with 4 cm in width. The surface temperature is 120 ^{o}C.

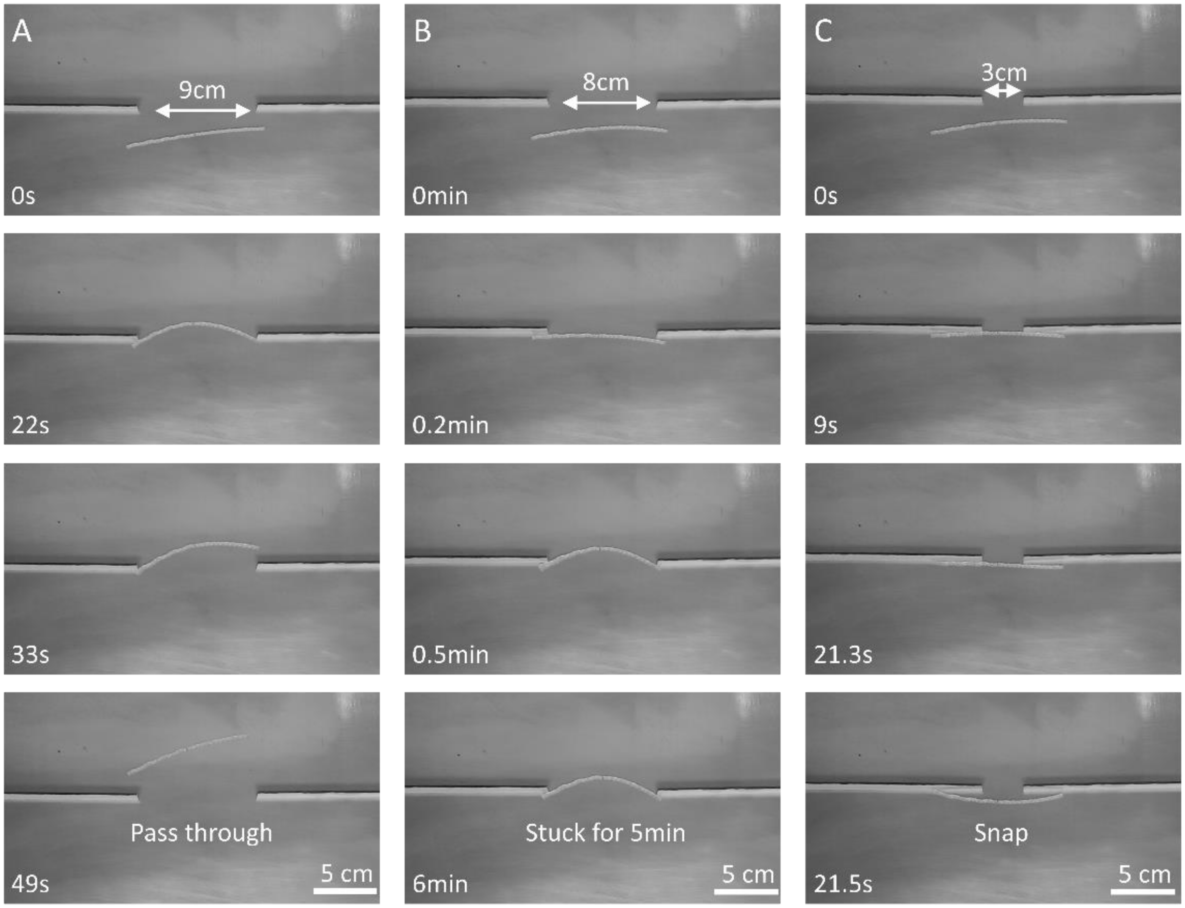

Figure S27. The hybrid sample interacts with narrow gaps constructed with thin wooden plates. (A) The sample can pass through a narrow gap with a width of 9 cm (~ 23.8% smaller than the sample length of 10.5 cm). **(B)** The hybrid sample is stuck for more than 5 min if the gap width is decreased to 8 cm. **(C)** The hybrid sample snaps when encountering a narrow gap with 3 cm in width. The surface temperature is 120 ^{o}C.

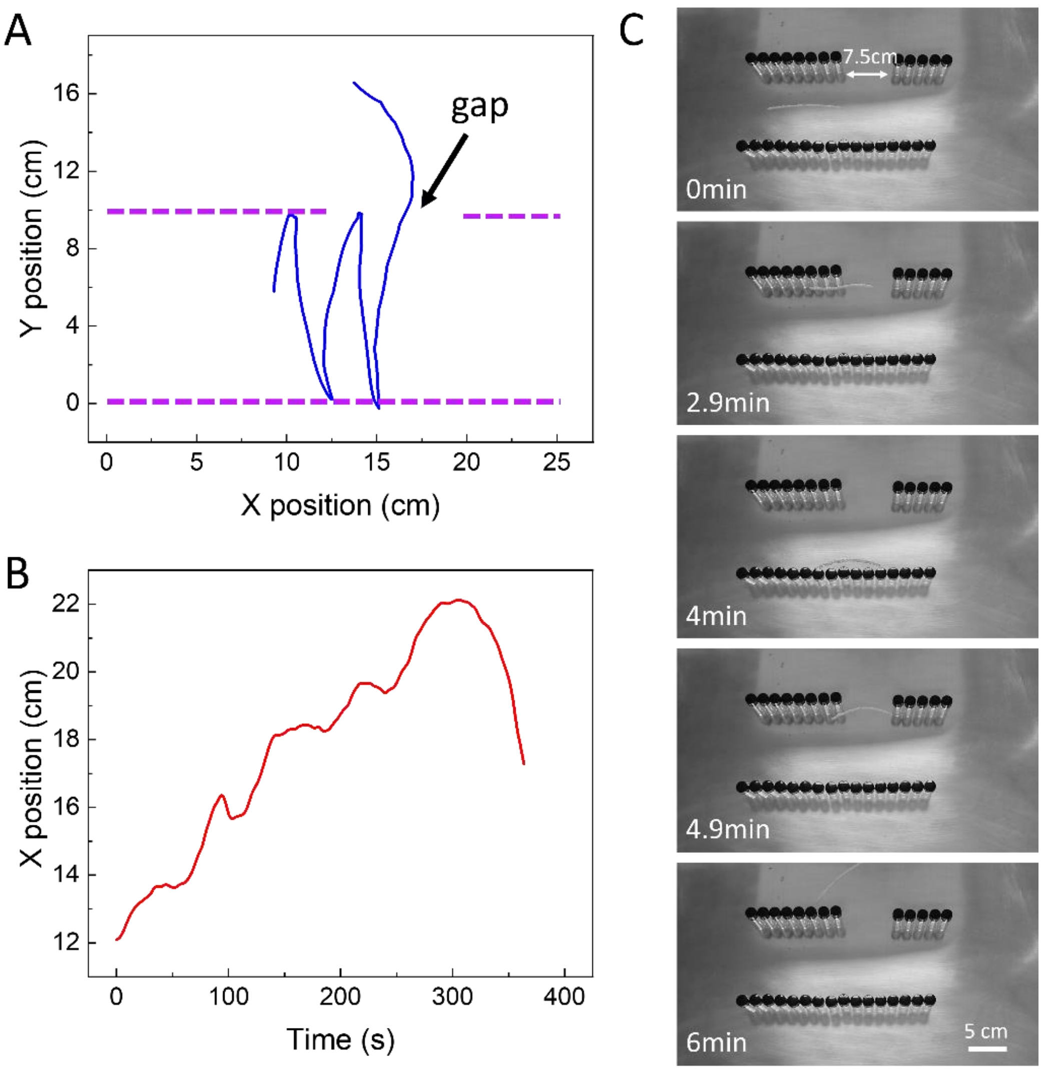

Figure S28. The hybrid sample self-escaping from a simple parallel confined space with a narrow gap with 7.5 cm in width. (A) The escaping trajectory. **(B)** The horizontal displacements (X positions) evolving with time. **(C)** The time lapses of the escaping process. The sample is in parallel to the walls initially. The surface temperature is 120 ^oC.

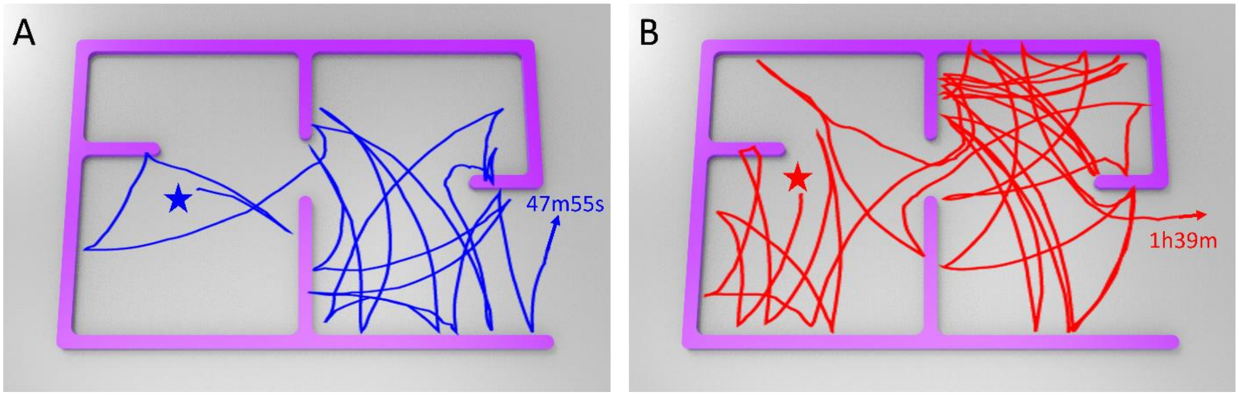

Figure S29. The paths for the hybrid sample escaping the maze in Figure 9D. (A) and **(B)** correspond to the cases starting from Location 2 and Location 3 in movie S10, respectively. The starting points are denoted as star symbols. The surface temperature is 120 ^{o}C.

Legends for Movie S1 to S11

Movie S1. The self-escaping capabilities of the twisted, helical, and hybrid ribbons in parallel confined space. The samples are placed at the same location with the same angle in the confined space initially. The hybrid ribbon can successfully escape from the confined space while the twisted and helical ribbons are trapped. The surface temperature is 120 ^{o}C.

Movie S2. The active turning behaviors of the twisted, helical, and hybrid ribbons during free self-rolling at different temperatures. For the hybrid ribbon, it can be either placed with its helical part to the left as shown in Fig. 2C or flipped with its helical part to the right as shown in Fig. 2D.

Movie S3. The FEA simulations of untwisting-induced self-turning of the twisted ribbon. First, a thermal-induced untwisting process in the ribbon without rolling is simulated. Then, an untwisting process with rolling is simulated at different temperatures.

Movie S4. The high-speed videos of the snap-through processes of the twisted, helical, and hybrid ribbons on a hot plate. The surface temperature is 120 ^{o}C.

Movie S5. The high-speed videos of the snap-through processes of the twisted, helical, and hybrid ribbons on sand. The temperature of the sand is 120 ^{o}C.

Movie S6. The hybrid ribbon autonomously escapes from mazes. The ribbon self-escapes three simple mazes with different starting positions. The escaping times can be largely different with different escaping trajectories. The surface temperature is 120 ^{o}C.

Movie S7. The twisted and helical ribbons are easily trapped in a simple maze. The surface temperature is 120 ^{o}C.

Movie S8. The hybrid ribbon autonomously escapes a maze on loose sand. The temperature of the sand is 120 ^{o}C.

Movie S9. The hybrid ribbon autonomously escapes two complex mazes. The escaping times are much longer than the simple mazes. The surface temperature is 120 ^{o}C.

Movie S10. The hybrid ribbon escapes from confined spaces and mazes with narrow gaps. The surface temperature is 120 ^{o}C.

Movie S11. Self-escaping of the hybrid ribbon from a maze with in-situ changing pattern. The maze changes the pattern every 5 minutes. The surface temperature is 120 ^{o}C.
